# Phage Endolysins as Promising and Effective Candidates for Use Against Uropathogenic *Escherichia coli*

**DOI:** 10.3390/v17040560

**Published:** 2025-04-13

**Authors:** Wojciech Wesołowski, Aleksandra Łukasiak, Sylwia Bloch, Kaja Kuligowska, Julia Neumann, Natalia Lewandowska, Emilia Węglińska, Grzegorz Węgrzyn, Bożena Nejman-Faleńczyk

**Affiliations:** 1 Laboratory of Biology and Biotechnology of Bacteriophages, Department of Molecular Biology, Faculty of Biology, University of Gdańsk, Wita Stwosza 59, 80-308 Gdansk, Poland; wojciech.wesolowski@phdstud.ug.edu.pl (W.W.); aleksandra.lukasiak@phdstud.ug.edu.pl (A.Ł.); sylwia.bloch@ug.edu.pl (S.B.); k.kuligowska.370@studms.ug.edu.pl (K.K.); natalia.lewandowska@phdstud.ug.edu.pl (N.L.); e.weglinska.166@studms.ug.edu.pl (E.W.); grzegorz.wegrzyn@ug.edu.pl (G.W.); 2 BNF—New Bio Force Ltd., Kartuska 420a, 80-125 Gdańsk, Poland; 3 Laboratory of Environmental Chemoinformatics, Faculty of Chemistry, University of Gdansk (UG), Wita Stwosza 63, 80-309 Gdansk, Poland; j.neumann.618@studms.ug.edu.pl

**Keywords:** bacteriophages, phage endolysins, uropathogenic *Escherichia coli* (UPEC), urinary tract infections (UTIs), phage therapy

## Abstract

The presented in silico and phylogenetic analysis of putative endolysins potentially produced by phages infecting uropathogenic *Escherichia coli* (UPEC) demonstrates their remarkable diversity. These proteins exhibit significant variations in sequence length, molecular weight, isoelectric point, and stability, as well as diverse functional domains determining their enzymatic activity, including lysin, lysozyme, hydrolase, amidase, and peptidase functions. Due to their predicted lytic properties, endolysins hold great promise in combating UPEC bacteria, including those within biofilms, which are often highly resistant to conventional treatments. Despite their potential, several challenges hinder the full utilization of endolysins. These include the relatively small number of identified proteins, challenges in the annotation process, and the scarcity of studies evaluating their efficacy in vitro and in vivo against Gram-negative bacteria. In this work, we emphasize these challenges while also underlining the potential of endolysins as an effective tool against UPEC infections. Their effectiveness could be significantly enhanced when combined with agents that disrupt the outer membrane of these bacteria, making them a promising alternative or complement to existing antimicrobial strategies. Further research is necessary to fully explore their therapeutic potential.

## 1. Introduction

Phage endolysins are the best-studied peptidoglycan-degrading enzymes [1,2]. These molecules are produced by bacteriophages during viral multiplication within the host. They belong to the lytic system that is responsible for the digestion of the bacterial cell wall from inside of the bacterium, thereby inducing fast osmotic lysis of host cells and the release of phage progeny into the environment [3,4,5]. When developed as a drug, endolysins must be stable, soluble, and able to degrade peptidoglycan from outside [5]. Taking into account the enzymatic activity and mechanism of the disruption of peptidoglycan, phage-derived endolysins can be subdivided into five different groups: (I) glucosaminidases (N-acetyl-β-D-glucosamidases), (II) lysozymes or muramidases (N-acetylmuramidases), (III) lytic transglycosylases, (IV) endopeptidases (L-alanoyl-D-glutamate endopeptidases), and (V) amidases (N-acetylmuramoyl-L-alanine amidases) [6]. The action of endolysins is frequently supported by other accessory proteins such as holins and pinholins. These proteins form pores in the cytoplasmic membranes and enable endolysins to enter and degrade the peptidoglycan layer [7,8].

Proteins functioning as endolysins during the phage life cycle have a globular or modular structure [9,10]. Moreover, their architecture differs between enzymes specific for Gram-positive and Gram-negative bacteria which is related to the different structures of the cell walls of these microorganisms [11]. The bacteriophage-derived endolysins of Gram-positive hosts have a molecular mass between 25 and 40 kDa and are composed of an N-terminal enzymatic activity domain (EAD) and a C-terminal cell wall-binding domain (CBD). However, some of these enzymes may also have more than one EAD and CBD domains in different orders. The EAD and CBD domains are connected by a short, flexible linker region [9,10,12,13]. The N-terminal EAD contributes to the simple enzymatic cleavage of different bonds in peptidoglycan structure, while C-terminal CBD can recognize and bind to specific ligand molecules on the cell wall [11,14,15]. Importantly, the modular structure of these Gram-positive phage-derived products allows protein engineering to improve the production process, enhance the activity against bacteria, widen the lytic spectrum of action of enzymes, and modify physicochemical properties of endolysins, such as stability and solubility [16,17,18]. In contrast, Gram-negative endolysins are mostly small, single-domain globular proteins with molecular mass between 15 and 20 kDa. Most of them contain only a single EAD domain, without a CBD [8,14]. Similarly to endolysins active against Gram-positive bacteria, some Gram-negative endolysins were found to have a modular structure. Examples include endolysins encoded by *Pseudomonas*, *Salmonella*, and *Burkholderia* bacteriophages [8,14]. Unfortunately, the presence of the outer membrane at the cell wall of Gram-negative pathogens makes the exogenous application of endolysins less attractive and effective compared to their counterparts produced by phages infecting Gram-positive bacteria. However, due to the huge problem of antibiotic resistance among Gram-negative bacteria, recently, the increasing attention of researchers has been devoted to developing new strategies for overcoming the obstacle of the outer membrane by Gram-negative targeted endolysins [14,19,20,21].

Antimicrobial resistance is a huge problem among UPEC that are responsible for urinary tract infections (UTIs). It is estimated that UPEC bacteria are responsible for approximately 50–65% of complicated cases and 75–85% of uncomplicated infections [22]. Although antibiotics are effective in treating uncomplicated UTIs, such a therapy becomes problematic for complicated infections, especially those caused by resistant bacterial strains. UPEC bacteria are hazardous due to their adhesive abilities, which enable the colonization of the urinary tract, as well as the formation of biofilm.

Biofilm formation is a crucial stage in the pathogenesis of UTIs, allowing UPEC bacteria to expand within the urinary tract effectively. Biofilm development is facilitated by the production of specific adhesins by UPEC, such as type 1 and P fimbriae, which recognize receptors on the surface of the host’s epithelial cells. This enables the bacteria to adhere to the epithelial cell layer, such as that of the bladder, and initiate the production of an extracellular matrix (EPS), which effectively protects the bacteria from the action of antibiotics and the host’s immune cells. Moreover, the biofilm contains persister cells, whose presence enables the immediate restoration/regrowth of the bacterial population after antibiotic treatment [23,24]. For this reason, biofilm removal is extremely challenging, and infection relapses are frequent. Biofilm can also be formed on abiotic surfaces, such as urinary catheters, making the treatment of UTIs in catheterized patients especially challenging. In the hospital environment, biofilm serves as a source of chronic infections, and the spread of resistant strains, due to the intensive transfer of genes between cells, which promotes the selection and dissemination of antibiotic resistance [25].

Bacteriophages and the endolysins they produce may help address the problem of biofilm removal due to their ability to go through the EPS structure and increase the exposure of bacterial cells to the action of the phages and their enzymes, as well as to applied antibiotics [11,26]. Phage therapy targeting biofilms, incorporating both phages and phage-derived enzymes, should address several challenges. The most significant are the following: insufficient number of characterized bacteriophages and phage proteins, the development of bacterial resistance to phages, and safety concerns. Importantly, there are no studies clearly indicating that phage lytic enzymes, including endolysins, induce the development of resistance similar to the one observed in the case of antibiotics or even phages. Therefore, great hopes are placed in them, among others, for combating UPEC. For this reason, in this review, we decided to summarize our knowledge about putative endolysins identified in the genomes of bacteriophages infecting UPEC bacteria to show their great application potential and indicate the main research problems. Importantly, we focus on both endolysins produced by phages specific only to UPEC, but also those derived from phages that, apart from UPEC, infect other types of *E. coli* bacteria. To our surprise, we could not find reports concerning in vitro and in vivo applications of these endolysins; thus, we focused mainly on the in silico characteristics of their sequences found in the GenBank database. We gathered 39 proteins annotated by authors as endolysins or proteins having endolysin domains and performed their detailed in silico analysis and phylogenetic searches. In addition, we summarized the literature reports on the use of endolysins derived from phages other than anti-UPEC to combat uropathogens, concentrating on the endolysin action against bacterial biofilms.

## 2. In Silico Analysis of Anti-UPEC Phage Proteins Annotated as Endolysins

This analysis considered 75 lytic anti-UPEC bacteriophages with DNA sequences found in the GenBank database on 15.12.2024. Table 1 summarizes the most important information and properties of the putative endolysins (39 in total) annotated by different authors in the genomes of tested bacteriophages [27,28,29,30,31,32,33,34,35,36,37,38,39,40,41]. In cases where the phage genomic sequence was available, but no annotations were made, we performed the annotation ourselves using the Pharokka version 1.6.1 [42]. All putative endolysins annotated by us were labeled as “self”, with the appropriate number indicating the open reading frame number assigned during the annotation process. For all proteins, annotated both by us and other scientists, we gathered data, such as the name of the protein, position of the gene in the virus genome, gene length, protein accession number, protein length, molecular weight, isoelectric point (pI), melting point (Tm), instability index, aliphatic index, grand average of hydropathy score (Gravy), name of the predicted domain, and its position in the protein sequence (Table 1). 

The data in Table 1 were obtained by us using several in silico tools. The Prot-Param module from the Biopython library was used to predict molecular weight, isoelectric point (pI), instability index, and Gravy score [43]. The DeepSTABp software was used to predict protein melting points [44]. The aliphatic index was calculated using a previously described formula [45]. A domain search was performed using InterProScan version 5.72-103 [46]. This tool integrated predictive information about proteins’ function from several partner resources (such as CDD, Coil, FunFam, Gene3D, Hamap, MobiDBLite, NCBIfam, Panther, Pfam, Phobius, PIRSF, PRINTS, ProSitePatterns, ProSiteProfiles, SMART, and SUPERFAMILY), giving an overview of the domains that a protein contains and families that it belongs to. All data generated by this program are collected in Supplementary Table S1. Notably, this analysis showed that some of the analyzed proteins do not have domains typical for phage endolysins, and they are described in more detail below.

In silico analyses indicated that genomes of 30 anti-UPEC bacteriophages contain genes encoding potential endolysins or proteins with endolysin domain [27,28,29,30,31,32,33,34,35,36,37,38,39,40,41]. In total, 39 putative enzymes were listed in Table 1. Interestingly, seven bacteriophages—EG1, LNA3, LNA5, SHAK9454, SHAK7163, SHAK7854, and SHAK7704 [27,33,39]—were identified to produce the internal virion proteins with endolysin domains (Table 1). In this group of 30 bacterial viruses, we can distinguish eleven bacteriophages that produce enzymes with lysozyme domains: 590B, Killian, NTEC3, LNA1, SHAK7693, SHAK7858, ACG-M12, UTEC10, Golestan, phiEc3, phiEc4 [27,28,29,30,31,32,33,34,35]; six phages with enzymes of amidase activity: 101118UKE1, 101120B1-2, 101120B2, 101136BS1, 22664BS1, 22664UKE3-2 [36]; six phages having endolysins with peptidase domain: ACG-C91, SHAK7704, SHAK7163, KMB14, KMB43 MLP1 [33,34,37,38]; EG1 phage [39] with enzyme having transglycosylase domain; and LNA4 phage [27] producing enzymes with hydrolase activity, as indicated in Table 1. The exceptions are seven internal virion proteins (YP_009795842.1, self_TCEH45, self_ZENB53, self_GXIJ6, self_DNYN53; self_RMKP62, and WVW77660.1) identified in phages SHAK7163, SHAK7704, SHAK7854, SHAK8454, LNA3, LNA5 [27,33], and five endolysins (WEU68017.1, WEU67833.1, UVD33329.1, UVD33388.1, and UVD33512.1) originated from phages Killian [35] and ZCEC14 [40], in which the in silico tools were unable to identify the domains typically characteristic of endolysins (Table 1, Supplementary Table S1).

As we mentioned above, the first considered group of endolysins are enzymes with domains of lysozymes (Table 1), and they are annotated in genomes of the 11 anti-UPEC phages: 590B, Kilian, NTEC3, LNA1, SHAK7693, SHAK7858, ACG-M12, UTEC10, Golestan, phiEc3, and phiEc4 [27,28,29,30,31,32,33,34,35]. Phages probably produce these potential N-acetylmuramidases to kill Gram-negative bacteria through targeted hydrolysis. As a result of such action, the integrity of peptidoglycan is interrupted and an imbalance of turgor pressure occurs, causing the lysis of bacterial cells [47,48,49]. The endolysins (YP_010749568.1, QYU43915.1, YP_009620148.1, YP_010749155.1, QVR48622.1, WWY66481.1, WVW77834.1, and self_ ZKRH133) of the phages phiEc3 [30], phiE4 [30], Golestan [32], NTEC3 [31], 590B [28], UTEC10 [29], SHAK7858 [33], and LNA1 [27] demonstrate the presence of endolysin and autolysin domains (cd00737, Table 1), and based on their domains, they are classified as lysozyme-like proteins or phage lysozymes (SSF53955, PF00959, respectively). These in silico-identified proteins have a predicted molecular mass of 8.73 to 18.59 kDa and an isoelectric point from 7.91 to 10.21 (Table 1). Interestingly, the endolysins (self_ ZKRH133, YP_006987885.1, YP_009620148.1, WVW77753.1, and WVW77754.1) encoded in genomes of the LNA1 [27], ACG-M12 [34], Golestan [32], and SHAK7693 [33] bacteriophages probably melt below 50 °C (Table 1). The remaining enzymes may denature at 50 °C and above (Table 1). The grand average of hydropathy (Gravy) of all proteins is below 0, indicating that they are soluble in water. The aliphatic index spans between 70.380 and 99.938, which indicates that these proteins are thermally stable and contain high amounts of hydrophobic amino acids. Moreover, the instability index of all of them is below 40, confirming these proteins’ stability (Table 1).

An interesting case among endolysins with a lysozyme domain is the endolysin (YP_006987885.1) synthesized by phage ACG-M12 [34]. It is an alkaline protein with a pI of 9.83, a melting temperature of 49 °C, and an aliphatic index of 99.938. This protein has a molecular weight of 17.63 kDa, is composed of 161 amino acids, seems to be stable as its instability index is equal to 33.838, and possesses the endolysin R21-like protein domain (cd16900, Table 1) that encompasses residues from 20 to 156. In addition to this, analyses from Phobius reveal that this protein contains a signal peptide spanning amino acids 1 to 27, and Hamap shows that it resembles SAR-endolysins (Supplementary Table S1) and may anchor to the membrane in an inactive form, which acts to prevent premature lysis of the infected host [50,51,52]. Another interesting protein with predicted lysozyme activity is the enzyme (WEU67899.1) encoded by the gene located in the DNA of the Killian phage [35]. This anti-UPEC virus possesses in its genome as many as four potential genes responsible for the production of endolysins. However, only one of them has the bacteriophage T4-like lysozyme domain (cd00735) that encompasses amino acid residues from 2 to 160 and is characteristic of N-acetylmuramidases (Table 1). The endolysin of the Killian phage has 164 amino acids, a molecular mass of 18.59 kDa, and the theoretical isoelectric point is estimated to be 9.59. Additionally, the critical melting point of the Killian-derived enzyme with the lysozyme activity equals 53 °C, the aliphatic index equals 88.049, and its instability index is 37.155, which, with the high value of the aliphatic index, may indicate its good stability (Table 1). In addition, it is worth emphasizing that all enzymes belonging to the group of proteins with lysozyme activity are involved in the peptidoglycan (GO:0009253) and cell wall macromolecule catabolic (GO:00016998) processes (Supplementary Table S1).

The second group of endolysins potentially produced by phages infecting UPEC bacteria are enzymes with an amidase domain (IPR002502; Supplementary Table S1). These proteins are probably produced by six viruses—22664UKE3-2, 101120B2, 101118UKE1, 101120B1-2, 101136BS1, and 22664BS1 [36]—and possess N-acetylmuramoyl-L-alanine amidase activity (PF01510; Table 1) and a peptidoglycan recognition protein (PGRP) domain (cd06583, Table 1). It is known that the peptidoglycan amidases act by breaking the amide bond that separates the glycan strand from the stem peptide located between the N-acetylmuramic acid and L-alanine residues [48,49]. Five of these proteins (QZI79721.1, QZI79903.1, QZI79784.1, QZI79844.1, QZI78611.1) are composed of 152 amino acids, their predicted molecular weight is approximately 17 kDa, and they are predicted to be unstable as the instability index remains over 40 (Table 1). The exception is amidase encoded by the 22664BS1 phage (QZI78465.1). This protein is slightly shorter (117 amino acids) and has a molecular mass of 13.16 kDa and an instability index equal to 50.611. Interestingly, all these enzymes possess similar isoelectric point values, ranging from 7.79 to 8.83, a theoretical melting point which ranges from 49 °C to 51 °C, and a Gravy score below 0, which indicates they are hydrophilic. Taking into account gene ontology, the analyzed endolysins with amidase properties are also involved in the peptidoglycan catabolic process (GO:0009253), as indicated in Supplementary Table S1.

In the analyzed group of putative endolysins encoded by phages infecting UPEC strains, there are also six proteins with peptidase activity (SSF55166, Table 1). These proteins (WPK27911.1, WQN06429.1, self_ RMKP5, WVW77668.1, YP_010844467.1, and YP_006987811.1) are encoded by genes located in the genomes of six phages: KMB14, KMB43, SHAK7704, SHAK7163, MLP1, and ACG-C91 [33,34,37,38]. The target of such proteins is a peptide that is made up of the L-lysine and D-alanine link [29,30]. Interestingly, the MLP1- [38] and KMB43- [37] derived peptidases (YP_010844467.1 and WQN06429.1) have the L-Ala-D-Glu peptidase-like domain (cd148945, Table 1), whereas ACG-C91- [34], KMB14- [37], SHAK7704- [33], and SHAK7163- [33] derived endolysins (YP__006987811.1, WPK27911.1, self_RMKP5, and WVW77668.1) have been identified to have the peptidase M15 domain (PF08291, Table 1). These enzymes’ length spans from 114 to 131 amino acid residues, and their molecular weights are between 12.59 and 14.96 kDa (Table 1). Moreover, the theoretical isoelectric point of potential peptidases is above 8.79, which indicates their alkaline nature. The predicted melting point of KMB14 putative peptidase (WQN06429.1) is 49 °C, which is the lowest predicted melting temperature, whereas the highest one is calculated for MLP1 putative peptidase (YP_010844467.1) and equals 57 °C. Importantly, most of these peptidases seem to be stable, besides KMB14- and MLP1- derived peptidases (WPK27911.1 and YP_010844467.1) with stability indexes of 40.639 and 47.821, respectively. Additionally, all of them show Gravy values below 0 and an aliphatic index above 65, indicating their thermal stability.

Interesting cases are also two putative endolysins encoded by genes located in the genome of the LNA4 bacteriophage [27]. Both of them seem to have hydrolase activity; however, in one of these two enzymes (annotated by us as self_PFXJ59), the chitinase class I domain (PF00182, Table 1) was found between amino acids 41 and 146. The presence of such a domain may suggest that this protein participates in the chitin catabolic process (GO: 0006032, Supplementary Table S1). This is a rather unusual feature for a phage, but a similar phenomenon has been observed previously in *Bacillus* infecting phage [53]. As indicated in Table 1, the endolysin has a molecular weight of 20.08 kDa, a pI of 9.3, and a Tm of 53 °C. Its instability index is low and equals 23.118, which demonstrates that the protein is stable. In addition, its aliphatic index is 88.098, a high value that also indicates the thermal stability of this protein. The Gravy index score equals -0.335, suggesting that this endolysin is more hydrophilic and soluble in water (Table 1). Interestingly, the second LNA4-derived endolysin (annotated by us as self_PFXJ204) probably has the cell wall hydrolase domain between amino acids 70 and 181 (PF07486, Table 1). This is a protein with a molecular mass of 21.65 kDa, a pI of 9.67, and a Tm of 41 °C. Its instability index equals 30.802, and the aliphatic index is 81.105, both suggesting the stability of this protein; and the Gravy score is −0.374 (Table 1).

It is worth emphasizing that anti-UPEC bacteriophages can potentially produce not only typical endolysins, but also the internal virion proteins with endolysin domains (Table 1). As we mentioned above, this phenomenon concerns seven bacterial viruses: SHAK9454, SHAK7163, SHAK7704, SHAK7854, EG1, LNA3, and LNA5 [27,33,39]. These enzymes are longer than the typical endolysins synthesized by bacteriophages. They are composed of over 1100 amino acids, and their molecular weight is estimated to be approximately between 121 and 145 kDa (Table 1). Moreover, their theoretical isoelectric point is within the range of about 5.61 to 6.58, and almost all of them seem to be stable. All of these proteins probably melt at temperatures between 41 °C and 42 °C and have an aliphatic index score between 77.892 and 79.459. Interestingly, among these bacteriophages [27,33], only the EG1-derived internal virion protein (YP_009795842.1) exhibits transglycosylase activity (PF01464, Table 1). This protein has the putative lytic transglycosylase (LT)-like domain between amino acids 28 and 144 (cd00254, Table 1). On the other hand, LNA3, LNA5-, SHAK7854-, and SHAK9454- derived internal virion proteins (self_GXIJ6, self_TCEH45, self_DNYN53, and self_ZENB53) are equipped with transmembrane regions (Supplementary Table S1), whereas the SHAK7163-, SHAK7704- derived proteins (WVW77660.1 and self_RMKP62) have coiled regions (Supplementary Table S1). Interestingly, the results of the Blast search indicated the highest similarity (100% query cover, 0.0 e-value, and 95.62% identity) of the EG1-derived protein to the DNA ejectosome component of *Yersinia* phage vB_YenP_WW2 and the outer membrane protein of *Yersinia* phage vB_YenP_WX1. On the other hand, Blast search against a non-redundant database of *E. coli* (taxid:562) records revealed that this protein is also similar to transglycosylase SLT domain-containing protein, with 100% query cover, 0.0 e-value, and 65.63% identity. In turn, LNA3-, LNA5-, SHAK9454-, and SHAK7854-derived proteins (self_GXIJ6, self_TCEH45, self_ZENB53, and self_DNYN53) are mostly common to internal virion protein D, with 100% query cover, 0.0 e-value, and identity varying from 96.76 to 98.46%. Lastly, SHAK7163- and SHAK7704-derived proteins (WVW77660.1 and self_RMKP62) are mostly similar to *E. coli* hypothetical proteins, also with 100% query cover, e-value equal to 0.0, and identity varying from 93.22% to 94.58%.

As mentioned before, in the Killian phage genome [35] there are four putative endolysins, but only one (WEU67899.1) with a phage lysozyme domain is described (Table 1). Other proteins, WEU67851.1 and WEU68017.1, have motifs not typical of endolysins, such as anaerobic ribonucleoside-triphosphate reductase domain (PF13597, Table 1) or a T4 RNase H motif (PF09293, Table 1), respectively. In the case of ZCEC14 phage [40], one of its putative endolysins (UVD33329.1) also possesses a T4 RNase H motif (PF09293, Table 1), and the second one (UVD33388.1) is considered to have a prohead protease inhibitor (IPR016594, Supplementary Table S1). Importantly, the identified domains suggest that these proteins may have other activities and are probably not endolysins.

Among these, we analyzed sequences of putative endolysins for the presence of antimicrobial peptides (AMPs) and found 39 such peptides with a high probability > 0.8, as indicated in Supplementary Table S2. The presence of such peptides indicates the lytic properties of analyzed endolysins against bacterial cells. Interestingly, some sequences of the identified peptides occur in more than one endolysin, which may suggest their evolutionary preservation.

The obtained results indicate a large diversity of domains present in endolysins and potentially high stability of the identified proteins. Moreover, we demonstrate that it is extremely important to thoroughly verify the annotated proteins in terms of functions they may perform.

## 3. Phylogenetic Analysis of Putative Endolysins Annotated in Genomes of Anti-UPEC Phages

**Figure 1:**
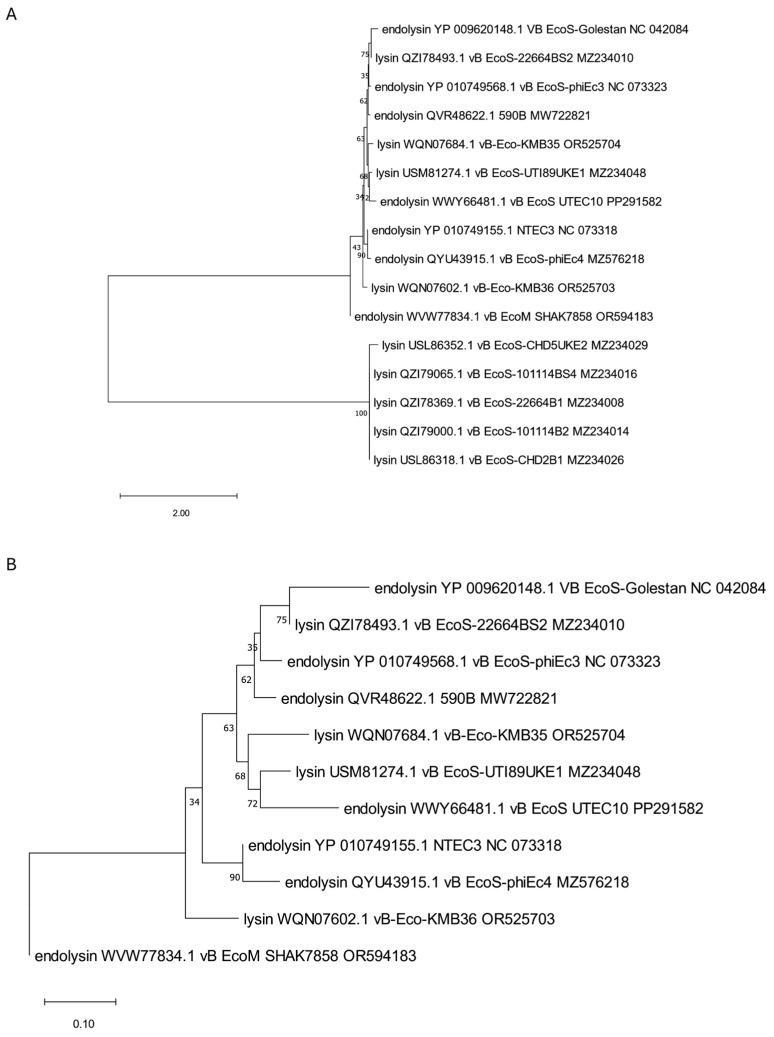
The phylogenetic tree is generated based on the first orthologous group determined by OMA and contains endolysins (YP_009620148.1, YP_010749568.1, QVR48622.1, WWY66481.1, YP_010749155.1, QYU43915.1, and WVW77834.1), with autolysin domains derived from phages Golestan, phiEc3, 590B, UTEC10, NTEC3, phiEc4, and SHAK7858, respectively. The tree was made as described in the text, using the maximum likelihood method with 1000 bootstrap replications. Panel (**A**) presents a tree with all grouped proteins. Panel (**B**) shows a modified tree, where proteins derived from phages CH5UKE2, 101114BS4, 22664B1, 101114B2, and CHD2B1 were removed to highlight the particular distances on the tree. The names indicated on the tree include the protein name and its accession number, followed by the phage name and its accession number.

**Figure 2:**
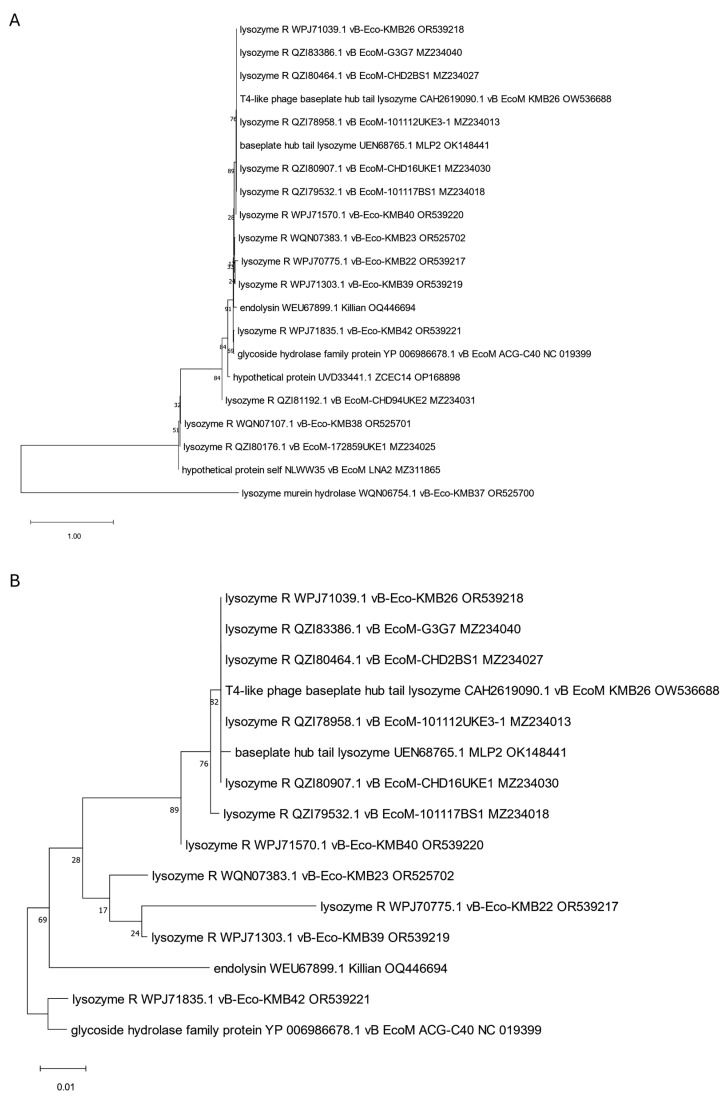
The phylogenetic tree is generated based on the second orthologous group estimated by OMA and contains a selected endolysin (WEU67899.1) of the Killian anti-UPEC phage. The tree was made using the maximum likelihood method with 1000 bootstrap replications. Panel (**A**) presents a tree with all grouped proteins. Panel (**B**) shows a modified tree, where proteins derived from phages KMB37, LNA2, 172859UKE1, KMB38, CHD94UKE2, and ZCEC14 were removed to highlight the particular distances on the tree. The names indicated on the tree include the protein name and its accession number, followed by the phage name and its accession number.

**Figure 3:**
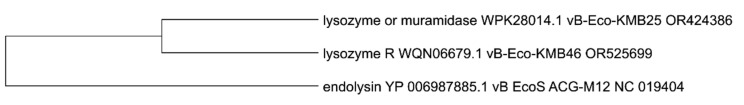
The phylogenetic tree is generated based on the third orthologous group determined by OMA and contains endolysin (YP_006987885.1) derived from phage ACG-M12. The tree was made using the maximum likelihood method. The names indicated on the tree include the protein name and its accession number, followed by the phage name and its accession number.

**Figure 4:**
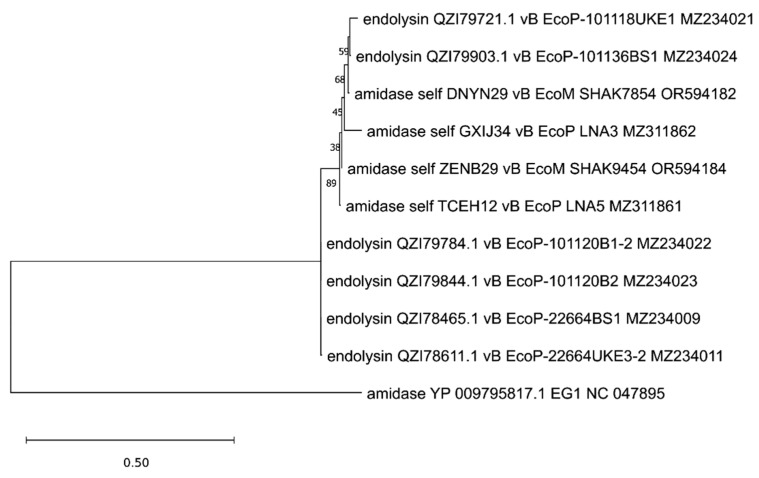
The phylogenetic tree is generated based on the fourth orthologous group estimated by OMA and contains endolysins with an amidase domain (QZI79721.1, QZI79903.1, QZI79784.1, QZI79844.1, QZI78465.1, and QZI78611.1). The tree was made using the maximum likelihood method with 1000 bootstrap replications. The names indicated on the tree include the protein name and its accession number, followed by the phage name and its accession number. The ‘self’-labeled proteins were annotated by us using Pharokka version 1.6.1 [42].

**Figure 5:**
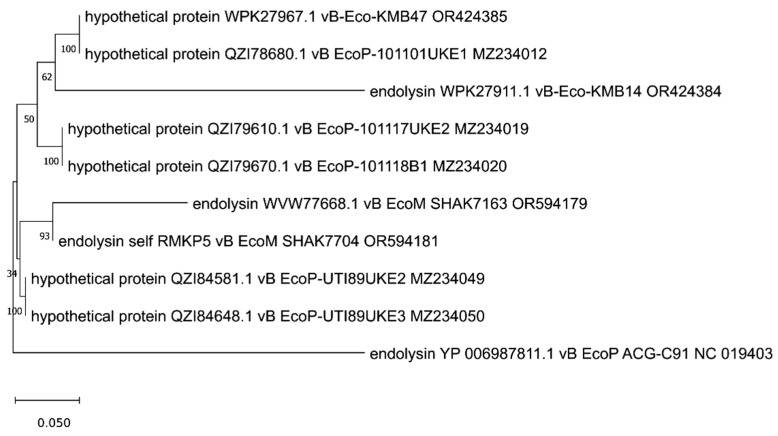
The phylogenetic tree is generated based on the fifth orthologous group determined by OMA and contains endolysins with peptidase domain (WPK27911.1, WVW77668.1, self_RMKP5, and YP006987811.1) derived from phages KMB14, SHAK7163, SHAK7704, and ACG-C91, respectively. The tree was made using the maximum likelihood method with 1000 bootstrap replications. The names indicated on the tree include the protein name and its accession number, followed by the phage name and its accession number. The ‘self’-labeled endolysin was annotated by us using Pharokka version 1.6.1 [42].

**Figure 6:**
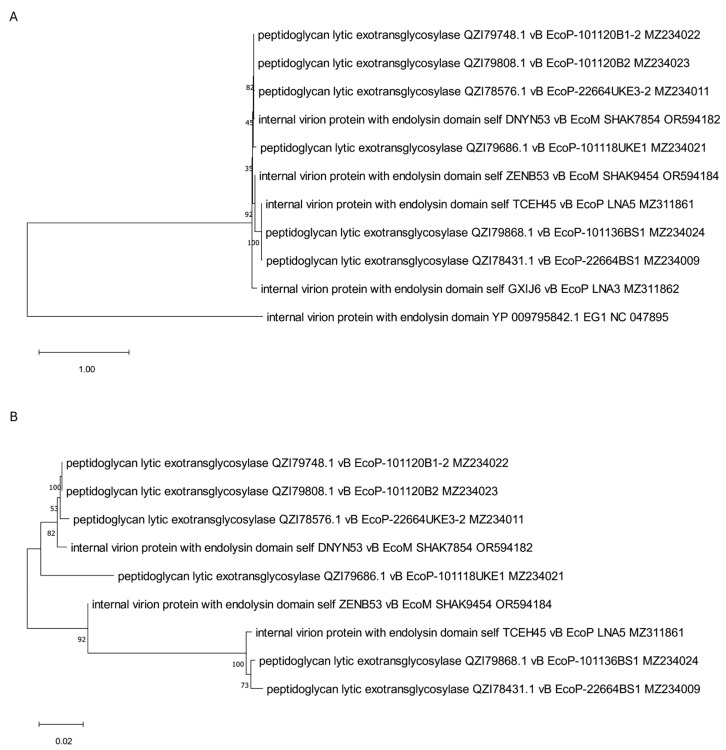
The phylogenetic tree is generated based on the sixth orthologous group determined by OMA and contains internal virion proteins with endolysin domain, such as EG-derived protein YP_009795842.1, and four other annotated and assigned by us: self_TCEH45, self_ZENB53, self_GXIJ6, and self_DNYN53. The tree was constructed using the maximum likelihood method with 1000 bootstrap replications. Panel (**A**) presents a tree with all grouped proteins. Panel (**B**) shows a modified tree, where proteins derived from phages EG1 and LNA3 were removed to highlight the particular distances on the tree. The names indicated on the tree include the protein name and its accession number, followed by the phage name and its accession number.

**Figure 7:**
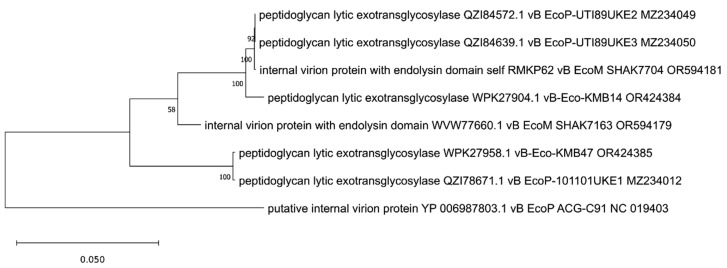
The phylogenetic tree is generated based on the seventh orthologous group established by OMA and contains internal virion proteins with a coiled-coil domain (WVW77660.1 and, annotated by us, self_RMKP62). It was made using the maximum likelihood method with 1000 bootstrap replications. The names indicated on the tree include the protein name and its accession number, followed by the phage name and its accession number.

**Figure 8:**
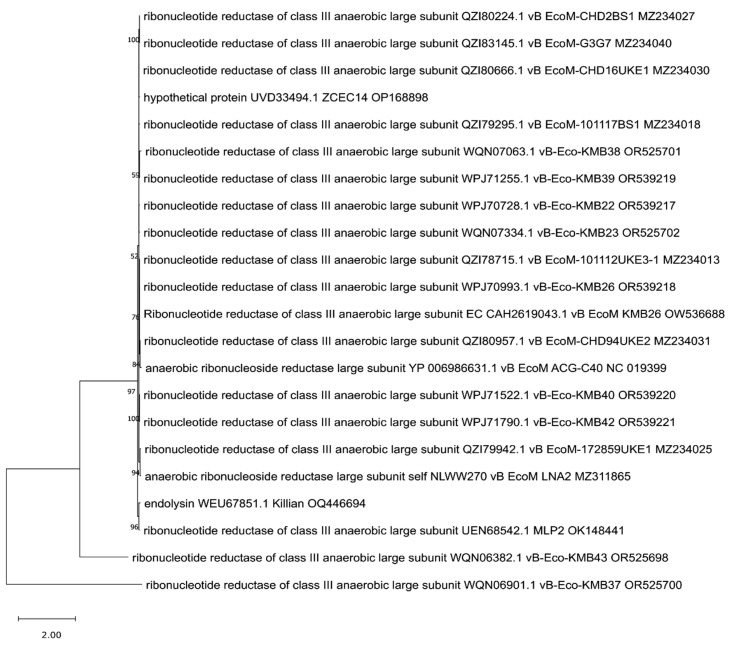
The phylogenetic tree is generated based on the eighth orthologous group established by OMA and contains putative endolysin of Killian phage (WEU67851.1). The tree was made using the maximum likelihood method with 1000 bootstrap replications. The names indicated on the tree include the protein name and its accession number, followed by the phage name and its accession number.

**Figure 9:**
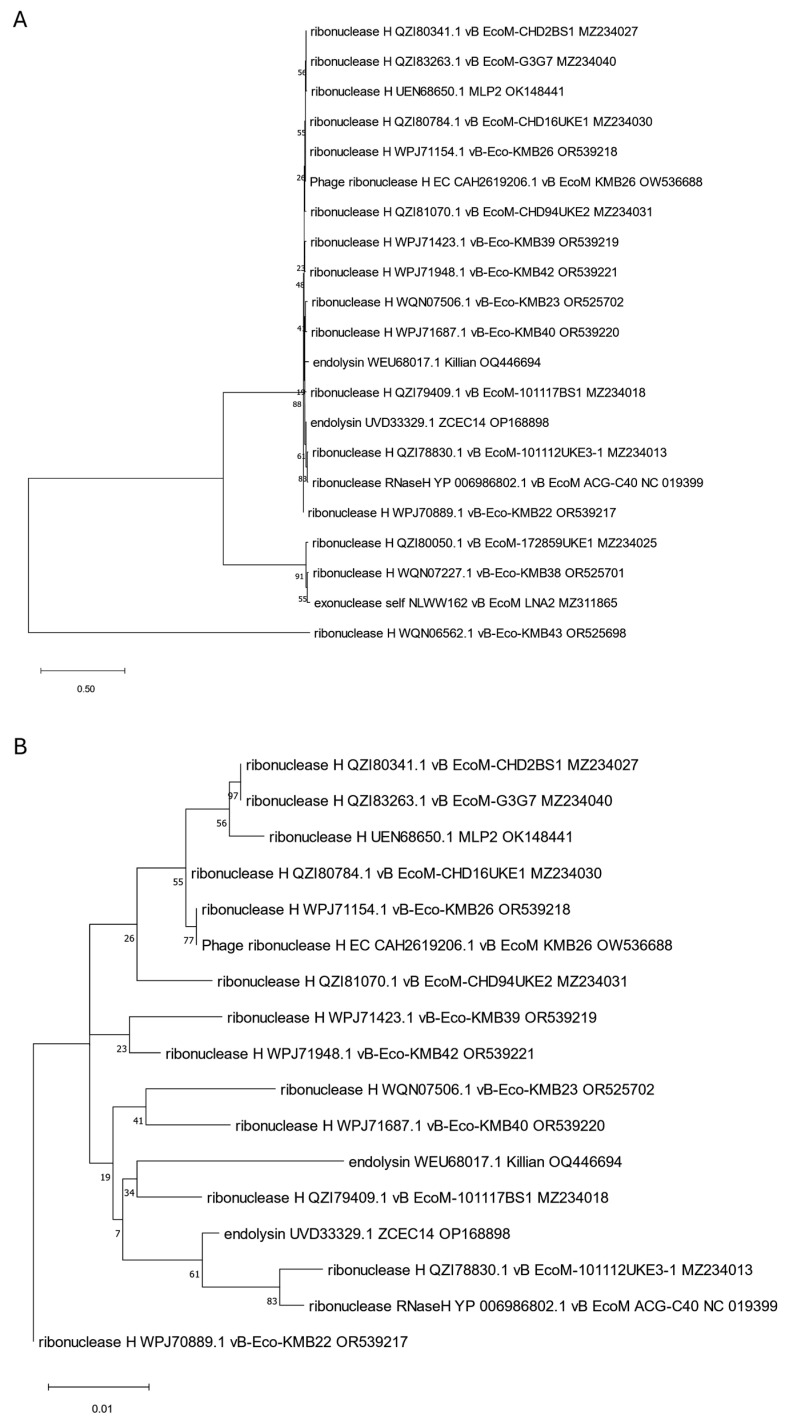
The phylogenetic tree is generated based on the ninth orthologous group determined by OMA and contains Killian’s putative endolysin no. WEU68017.1 and ZCEC14 putative endolysin no. UVD33329.1. It was constructed using the maximum likelihood method with 1000 bootstrap replications. Panel (**A**) presents a tree with all grouped proteins. Panel (**B**) shows a modified tree, in which proteins of phages KMB43, LNA2, KMB38, and 172859UKE1 were removed to highlight the particular distances on the tree. The names indicated on the tree include the protein name and its accession number, followed by the phage name and its accession number.

**Figure 10:**
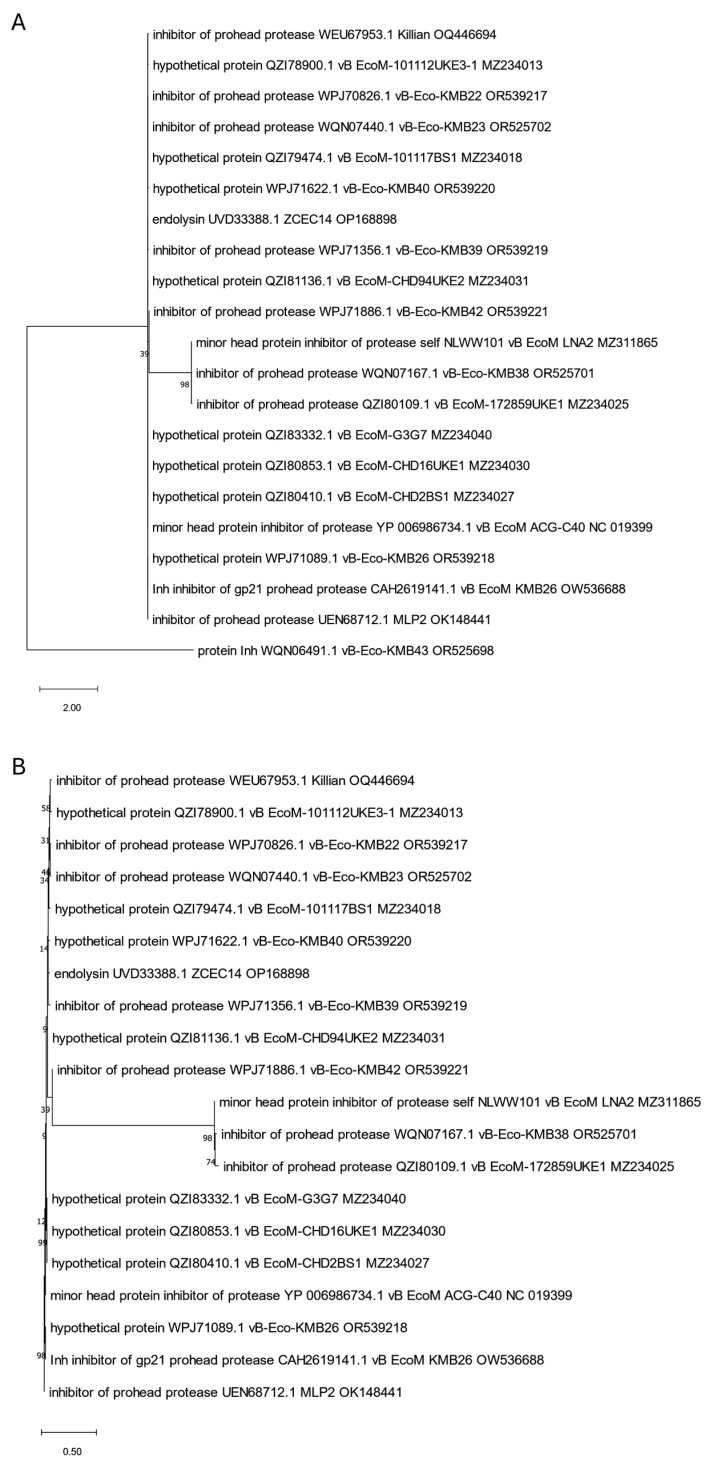
The phylogenetic tree is based on the tenth orthologous group identified by OMA and includes the ZCEC14-derived putative endolysin (UVD33388.1). It was constructed using the maximum likelihood method with 1000 bootstrap replications. Panel (**A**) displays the tree with all grouped proteins, while panel (**B**) presents a modified version in which the phage KMB43 protein was removed to emphasize specific distances within the tree. The names on the tree consist of the protein name and its accession number, followed by the phage name and its accession number.

**Figure 11:**
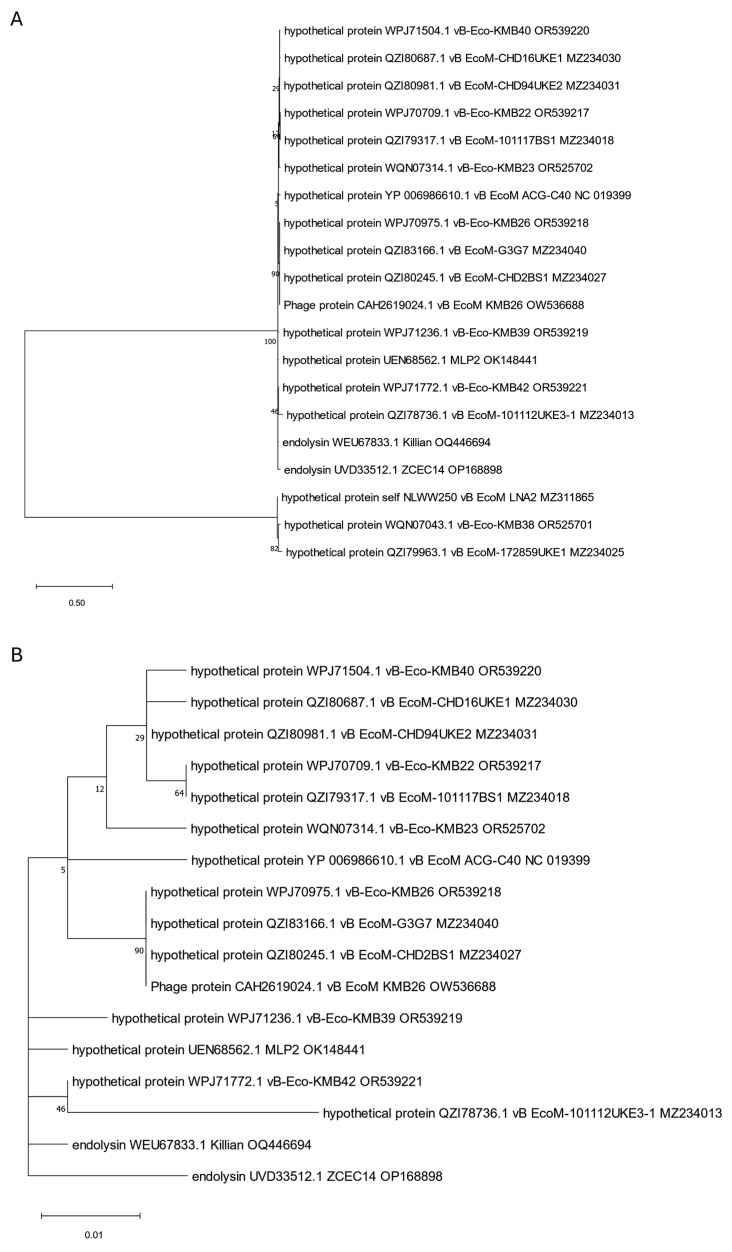
The phylogenetic tree is generated based on the eleventh orthologous group determined by OMA and contains two putative endolysins of Killian and ZCEC14 phages (WEU67833.1 and UVD33512.1). It was made using the maximum likelihood method with 1000 bootstrap replications. Panel (**A**) presents a tree with all grouped proteins. Panel (**B**) shows a modified tree, in which the proteins of phages LNA2, 172859UKE1, and KMB38 were removed to highlight the particular distances on the tree. The names indicated on the tree include the protein name and its accession number, followed by the phage name and its accession number.

The analyzed putative endolysins differ significantly, as indicated in the previous paragraph. To establish their common evolutionary origin, the OMA [54] software version 2.6.0 was used to determine 11 orthologous groups for all 39 annotated endolysins and other proteins identified in 75 genomic sequences of anti-UPEC phages found in the NCBI database on 15.12.2024 [27,28,29,30,31,32,33,34,35,36,37,38,39,40,41]. Then, based on such orthologous groups containing endolysin sequences, maximum likelihood trees were prepared using IQ-TREE [55]. The alignments of nucleotide sequences were performed using Clustal Omega version 1.2.4 [56], and the MEGA [57] program was used to perform visualization with midpoint rooting. This analysis allowed us to generate 11 separate trees (Figure 1, Figure 2, Figure 3, Figure 4, Figure 5, Figure 6, Figure 7, Figure 8, Figure 9, Figure 10 and Figure 11), which are discussed in detail below. In addition, we also identified proteins that were not annotated by the authors as endolysins, but turned out to be closely related to them and may have similar functions. Importantly, phylogenetic trees were not obtained for some of the described endolysins (WVW77754.1, WVW77753.1, self_ZKRH133, WQN06429.1, YP_010844467.1, self_PFXJ59, and self_PFXJ204) due to the lack of their sequence similarity to other annotated proteins.

For convenience, we gathered all potential endolysins that appeared in our phylogenetic analysis in Supplementary Table S3. These were annotated differently by the authors (e.g., lysins, lysozymes, amidases, hydrolases, or hypothetical proteins). We performed analogous analyses for them, similar to those conducted for the originally annotated endolysins in Table 1. The results are presented in Supplementary Table S3. In addition, we performed a detailed domain search for all these proteins using InterProScan version 5.72-103 [47] and listed the obtained data in Supplementary Table S4. Among these, we analyzed the sequences of these proteins for the presence of AMPs and found 42 such peptides with high probability (>0.8). Interestingly, a few sequences of these peptides seem to be conserved among found proteins, as they repeat and occur in unchanging form in different putative enzymes (Supplementary Table S5).

The tree presented in Figure 1 includes almost all endolysins (YP_009620148.1, YP_010749568.1, QVR48622.1, WWY66481.1, YP_010749155.1, QYU43915.1, and WVW77834.1) with endolysin and autolysin domain (cd00737, Table 1) beyond endolysin from phage LNA1 [28] which, despite having the same domain type, has no common evolution origin. Interestingly, all endolysins included on the tree are closely related to proteins annotated as lysins in other anti-UPEC phages and probably act as their endolysins (Supplementary Tables S3 and S4).

Another two phylogenetic trees were prepared for the endolysins no. WEU67899.1 and YP_006987885.1 that have lysozyme domains and are derived from Killian [36] and ACG-M12 [35] phages, respectively (Figure 2 and Figure 3). Within endolysin WEU67899.1 of the Killian phage, we identified, using different programs, three similar domains—a T4-like lysozyme domain (cd00735), a phage lysozyme (PF00959), and a lysozyme-like domain (SSF53955)—as indicated in Table 1. Phylogenetic analysis shows this protein is closely related to other phage proteins annotated as lysozyme R (Figure 2A,B). In turn, the ACG-M12-derived endolysin with a R21-like domain (cd16900), a phage lysozyme (PF00959), and a lysozyme-like domain (SSF53955) are grouped on the tree (presented in Figure 3) with proteins WPK28014.1 and WQN06679.1 annotated as lysozymes in genomes of KMB25 and KMB46 phages [37] that can serve as potential endolysins in these phages (Supplementary Tables S3 and S4).

Lastly, there are two SHAK7693- derived [33] unique endolysins (WVW77754.1 and WVW77753.1) with function confirmed by InterProScan but showing no similarity to other proteins.

Another tree, presented in Figure 4, encompasses endolysins containing amidase domain (PF01510, Table 1). All six originally annotated endolysins [36] with this domain (QZI79721.1, QZI79903.1, QZI79784.1, QZI79844.1, QZI78465.1, and QZI78611.1) are grouped with other proteins annotated as amidases, which can serve as potential endolysins for these phages and are indicated in Supplementary Tables S3 and S4. Importantly, four of the analyzed endolysins (QZI79784.1, QZI79844.1, QZI78465.1, and QZI78611.1) are almost identical in sequence, while the two others (QZI79721.1 and QZI79903.1) show a little disparity.

The next tree, presented in Figure 5, is based on endolysins with the domain classified as peptidase M15 (PF08291, Table 1). Interestingly, three of the four analyzed endolysins (WPK27911.1, WVW77668.1, and YP006987811.1) derived from phages KMB14 [37], SHAK7163 [33], and ACG-C91 [34], respectively, have longer branch lengths, indicating more genetic changes. This implies faster evolution of these proteins compared to hypothetical proteins. On the other hand, domain analysis conducted with InterPro shows that these hypothetical proteins have common peptidase M15 and hedgehog/DD-peptidase domains with the analyzed endolysins (Supplementary Tables S1 and S4). Unfortunately, two other endolysins (WQN06429.1 and YP_010844467.1) with peptidase domain no. PF13539, derived from phages KMB43 [37] and MLP1 [38], respectively, were not collected into the orthologous group by OMA, so they were not included in this analysis.

A separate tree was created, including proteins annotated as internal virion proteins with the endolysin domain (Figure 6). Importantly, all these proteins are significantly bigger than typical phage endolysins and present molecular weights from 120 to 145 kDa (Table 1). Among them, one interesting protein (YP_009795842.1) derived from EG1 phage [39] contains the SLT domain (PF01464, Table 1). This protein is grouped on the tree with four other internal virion proteins (annotated and assigned by us: self_GXIJ6, self_TCEH45, self_ZENB53, and self_DNYN53) originating from phages LNA3, LNA5, SHAK9454, and SHAK7854 [27,33] and showing transmembrane regions in their sequence (Supplementary Table S1). All of them seem to be closely related to phage proteins annotated as peptidoglycan lytic exotransglycosylases. However, the internal virion protein (YP_009795842.1) of EG1 phage [39] is the protein least related to others. Importantly, apart from internal virion proteins, the phages analyzed here have in their genomes genes encoding amidases, which were not annotated directly as endolysins but may serve such functions, as indicated in Supplementary Tables S3 and S4.

Two other internal virion proteins (WVW77660.1 and, annotated by us, self_RMKP62) derived from phage SHAK7163 [33] and phage SHAK7704 [33], respectively, have been surprisingly classified by OMA into another orthologous group. The generated tree (Figure 7) shows that these two proteins with established coiled-coil structural motifs (Supplementary Table S1) are also closely related to proteins annotated as peptidoglycan lytic exotransglycosylases, but the least related protein, in this case, is a putative internal virion protein originating from phage ACG-C91 [34]. Importantly, except for internal virion proteins, the typical phage endolysins (WVW77668.1 and self_RMKP5) were also identified in genomes of phages SHAK7163 and SHAK7704 (Table 1). This may suggest that, although internal virion proteins may degrade peptidoglycan similarly to endolysins, they should not be considered classical endolysins participating in the lysis of bacterial cells that allow phage particles to release [58].

Since more than one endolysin was identified in Killian [35] and ZCEC14 [40] phages, we suspected that some of them might be annotated incorrectly and decided to take a closer look at the phylogenetic positions of all of them to clarify this. We created trees for them based on the orthologous group identified by OMA. We observed that one of the Killian putative endolysins (WEU67851.1), with predicted anaerobic ribonucleoside-triphosphate reductase feature (PF13597, Table 1), was grouped and closely related to many proteins annotated as large sub-units of ribonucleotide reductases (Figure 8). Another Killian putative endolysin (WEU68017.1), as well as a ZCEC14 putative endolysin (UVD33329.1) with a predicted 5′-3′ exonuclease, N-terminal resolvase-like domain (PF02739, Table 1), and T4 RNase H motif (PF09293, Table 1), turned out to be related to many ribonucleases H and one exonuclease (Figure 9). Another ZCEC14-derived putative endolysin (UVD33388.1), with prohead protease inhibitor (IPR016594, Supplementary Table S1) was, in turn, mostly related to inhibitors of head proteases and a few hypothetical proteins (Figure 10). Finally, the last two putative endolysins of Killian and ZCEC14 phages (WEU67833.1 and UVD33512.1) were grouped on the tree with many hypothetical proteins (Figure 11). Importantly, no specific endolysin domains were found within their sequences (Table 1).

Based on the presented results of phylogenetic analysis, we suggest that some proteins of anti-UPEC phages originally annotated as endolysins may play other roles. Phages in which more than one endolysin was annotated were particularly suspicious to us, and these doubts were clarified above. Our assumptions were confirmed because some of the originally annotated phage endolysins were found to be closely related to proteins with completely different functions (Figure 8, Figure 9 and Figure 10). In addition, we found that proteins annotated as internal virion proteins with endolysin domains differ significantly from typical endolysins in size and domain content and are known to play a different role than typical endolysins. As reviewed by Rodriguez-Rubio [58], virion-associated peptidoglycan hydrolases (VAPGHs) are enzymes that locally degrade the peptidoglycan of the bacterial cell wall during the beginning of the phage infection. In contrast to endolysins that mediate the lysis of the host bacteria at the end of the lytic cycle, the action of VAPGHs generates a small hole through which the phage tail tube crosses the cell envelope to inject the phage genetic material at the beginning of the infection cycle.

On the other hand, phylogenetic analysis performed here allowed us to identify other putative endolysins that were initially annotated by the authors as lysins, lysozymes, amidases, hydrolases, or hypothetical proteins but which may serve as phage endolysins. All of them are gathered in Supplementary Tables S3 and S4 and are shown to have AMPs in their sequences (Supplementary Table S5).

Undoubtedly, the annotation of phage endolysins is not an easy process due to the diversity of domains and motifs present in their sequences. Nevertheless, such analysis should be performed more carefully and be verified by other methods, including phylogenetic position determination. Such an approach may allow us to obtain more reliable results. In addition, the nomenclature of endolysins should be standardized. Currently, the name of an annotated protein is frequently assigned based on the identified domains and not the function it probably performs in the phage life cycle. Endolysins are very problematic in this respect as some authors annotate these proteins according to their purpose, and others according to the identified domain, which introduces confusion and difficulties in their identification.

Importantly, phage endolysins might also be grouped based on the similarity to endolysins produced by model phages, simply as (I) lysozymes of T4-like phages, (II) endopeptidases of T5-like phages, (III) amidases of T7-like phages, (IV) small peptidases of SP6-like phages, and (V) lysosymes of lambda-like phages. Considering this, we decided to perform detailed characteristics of model endolysins that confirmed the presence of appropriate domains and predicted their high stability (Supplementary Table S6). Then, we analyzed all the endolysins tested in this work in terms of their similarity to endolysins of model phages. Interestingly, we revealed that sequences of analyzed proteins were similar to model endolysins at varied levels, from 0.85% to 96.97% (Supplementary Table S7). However, the similarity at the level of about 1% was calculated for internal virion proteins, which, as we considered above, do not belong to typical endolysins. Among the classical endolysins, the protein originated from the LNA4 phage was the least similar to any reference endolysin, showing only 4.32% and 9.95% similarity to endolysins of phage T5 and T4, respectively. Importantly, we did not find any endolysin that was similar to λ phage protein in more than 5%. This observation coincides with the fact that only endolysins derived from lytic phages are considered in this work. Interestingly, 18 of the studied proteins showed very high sequence similarity (> 90%) to the reference endolysins. In total, a similarity above 70% was noted in 41 cases. For details, please see Supplementary Table S7.

## 4. In Vitro and In Vivo Testing of Endolysin Properties, Including Their Capability to Eradicate Biofilms

The formation of bacterial biofilms is a huge problem in UTI treatment. The harsh conditions within the urinary system, such as the flushing of cells during urination, force bacteria to develop strong adhesion mechanisms. In addition to the walls of the human bladder, bacterial biofilms can also form on medical surfaces, like catheters, leading to catheter-associated urinary tract infections (CAUTIs) [25]. Importantly, bacteria living in biofilm structures are frequently highly resistant to antibiotics; thus, addressing biofilms is crucial in the treatment of urinary tract infections.

Phage enzymes, including endolysins, show great promise in combating infections caused by antibiotic-resistant bacterial strains. Research on bactericidal proteins targeting Gram-negative bacteria is challenging due to the presence of the additional outer membrane that complicates access to peptidoglycan, the substrate for these enzymes [48]. The situation becomes even more complex when biofilms are involved, as the matrix serves as an additional layer of protection for bacterial cells, making it difficult for antibacterial agents to penetrate it [59]. Such challenges forced the necessity of searching for new effective treatment options. An interesting direction seems to study the effects of phage endolysins on biofilm-forming Gram-negative bacteria, also in combination with bacteriophages or antibiotics. One significant advantage of using phage enzymes is the lower likelihood of bacteria developing resistance to them, unlike traditional bacteriophage or antibiotic applications, where resistance cannot be completely ruled out [60]. The literature contains numerous reports on the activity of endolysins against bacteria from the ESKAPE group, such as endolysins LysAB1245 [61], LysJEP8 [62], and LysPA26 [63], targeting biofilms produced by *Pseudomonas aeruginosa*, or endolysins LysAm24, LysAp22, LysECD7, and LysSi3 [64] against biofilms of *Klebsiella pneumoniae*, *Acinetobacter baumannii*, and *P. aeruginosa*. However, there are relatively few reports on endolysins targeting uropathogens, particularly UPEC strains.

In the study conducted by Kajsikova et al. [65], the impact of an endolysin named EN572-5 on clinical uropathogenic isolates, primarily Gram-positive *Streptococcus agalactiae* bacteria, was investigated. The study also included four Gram-negative UPEC strains. The endolysin EN572-5, derived from *S. agalactiae* prophage KMB-572-E, exhibited lytic activity against 32 strains of *S. agalactiae*, averaging an impressive 83% effectiveness. The authors emphasized that no correlation could be observed between the enzyme’s activity and the type of bacterial strain, confirming that phage enzymes are characterized by a broader spectrum of activity, in contrast to bacteriophages. On the other hand, endolysin EN572-5 showed activity against UPEC strains at low levels of 16-20%. However, its action was tested without the addition of compounds that destabilize the outer membrane—a primary barrier for this type of enzyme. This highlights how the outer membrane hinders the enzyme’s access to peptidoglycan in Gram-negative bacteria. Importantly, this enzyme was also inactive against bacteria playing a protective role, like *Lactobacillus* spp., whose absence as a result of antibiotic treatments for UTIs can lead to complications such as bacterial vaginosis [65].

In the study conducted by Fursov et al. [66], the focus was on comparing the effects of the coliphage endolysin LysECD7 and amikacin against biofilm formation by a clinical multidrug-resistant strain of *K. pneumoniae* Ts 141-14, isolated from a patient’s urine. It was demonstrated that both the endolysin and the antibiotic exhibited similar efficacy against forming biofilms in vitro. However, the enzyme showed greater bactericidal activity against mature biofilm, whose density was decreased by 60% and 68% compared to the control group. Notably, the concentrations used in this work (1000 and 3000 µg/mL) were highlighted by the authors as a potential limitation for the therapeutic application of the enzyme. The results of the in vivo experiments, however, indicated significant therapeutic potential for this endolysin. The researchers used rats implanted with specially designed chambers containing a nitrocellulose membrane filled with a suspension of *K. pneumoniae* Ts 141-14 cells. On the fourth day post-infection, treatment was initiated with LysECD7 administered daily for seven days, delivering 50 µg. This resulted in a reduction in biofilm biomass as early as two days after starting the therapy. On the eighth day of treatment, the colony count decreased from 1000 viable bacterial cells to an average of 58 cells. Amikacin produced a similar effect, although in this case, the number of bacteria increased compared to the fifth day, when the first measurement was taken [66].

Interestingly, in another study [67], the activity of three different endolysins derived from *Pseudomonas* phages was examined against various bacterial species, including clinical isolates obtained from patients with UTIs. The tested group included uropathogens such as *E. coli* strain 531, *K. pneumoniae* strains identified as ESBL (extended-spectrum beta-lactamases) producers, and *P. aeruginosa* strain 471. Additionally, other bacterial strains belonging to the ESKAPE group were also included. Notably, one of these endolysins, named PlyKp104 [66], exhibited the best bactericidal activity and a broad spectrum against Gram-negative bacteria. Its action reduced the number of viable cells (CFU/mL) of *P. aeruginosa*, *K. pneumoniae*, *E. coli*, and *A. baumannii* strains by five or six orders of magnitude in most tested variants. The authors did not use additional permeabilizing agents and attributed the activity of the PlyKp104 endolysin against Gram-negative bacteria to the presence of a positively charged C-terminal region, which interacts with the outer membrane. This natural ability to kill Gram-negative bacteria undoubtedly highlights its great potential in combating Gram-negative pathogens, including uropathogenic strains. The knowledge about PlyKp104 biofilm-eradicating capabilities is highly valuable and the researchers tested its lytic activity in vivo using a mouse skin wound infection model with *K. pneumoniae*. A single application of 300 μg of PlyKp104 was used, and after three hours, a reduction in the number of viable bacterial cells by two orders of magnitude was observed [67].

An interesting approach to overcoming the outer membrane of Gram-negative bacteria involved the use of a modified endolysin Lysep3 [68] with additional hydrophobic amino acid residues. The study examined the native endolysin and variants with added hydrophobic residues at the C-terminus, consisting of three, five, seven, and twelve residues. Enzymes with 12 residues were tested in two variants differing in amino acid composition. All proteins were applied externally. Endolysins with a higher number of hydrophobic residues exhibited greater bactericidal activity against *E. coli* bacteria compared to the unmodified enzyme. The activity increased with the number of residues, while no differences were noted regarding amino acid composition, as the degree of hydrophobicity was the same [68]. Meanwhile, Hasan et al. [69] used lactic acid in combination with two endolysins, LysEP114 and LysEP135, to combat biofilm formation by the *E. coli* KCCM 40405 strain. When applied together, the enzymes and lactic acid demonstrated significantly greater antibacterial and antibiofilm effects than when used separately. The numbers of biofilm cells were reduced by more than three and five logs for LysEP114 and LysEP135, respectively. The authors highlight an important observation that lactic acid’s ability to permeabilize the outer membrane of Gram-negative bacteria gives it an advantage, particularly for applications such as food safety control [69]. In the field of food safety, significant attention is focused on the *E. coli* O157:H7 strain, a member of the STEC (Shiga toxin-producing *E. coli*) group, which causes severe gastrointestinal symptoms and is often linked to contaminated fruits and vegetables. It has been demonstrated that the endolysins LysECP26 [70], LysPECP14, and LysPECP20 [71] can effectively kill bacterial cells after pre-treatment with EDTA. However, the referenced studies examined only the bactericidal effect on liquid cultures, and the biofilm-disrupting potential of these enzymes remains unknown. Polymyxin B or cecropin A fused at the N-terminus, which is an antibacterial peptide, are capable of disrupting the structure of the outer membrane and have also been shown to effectively eradicate planktonic cells of *K. pneumoniae*, *P. aeruginosa*, *A. baumannii*, and *E. coli* in combination with the endolysin LysKP213 both in vitro and in the *Galleria mellonella* model [72]. A particularly interesting fact is that the mentioned endolysin, LysKP213, is highly thermostable. Its lytic activity remains at the level of 44.4% after 20 h of incubation at 95 °C, while after 30 min of autoclaving at 121 °C, it retains activity at up to 57.5%. This is valuable information regarding the potential applications of this protein in biotechnology, the medical industry, and food preservation [72].

In the context of the safety of therapies using endolysins against Gram-negative bacteria, including *E. coli*, the findings presented by Hwang et al. [73] are particularly intriguing. The researchers demonstrated that the endolysin LysPA90, encoded by the bacteriophage PBPA40, which infects *P. aeruginosa* strains, affects the motility of *E. coli* AIEC strain (adherent invasive *E. coli*). Sub-inhibitory concentrations of this protein caused stress in bacterial cells and increased the expression of genes responsible for flagella biosynthesis. This resulted in enhanced adhesion to intestinal epithelial cells of the Caco-2 line. The increased adhesion of bacterial cells was accompanied by elevated levels of proinflammatory cytokines in eukaryotic cells [73]. Therefore, in addition to studying the activity of enzymes against pathogens, very thorough and precise research is necessary to assess the safety of such therapies. The study mentioned earlier demonstrates that endolysins, even if they do not exhibit bactericidal activity, can influence the bacterial phenotype in ways that may be unfavorable for the patient.

The growing antibiotic resistance is a global problem that creates an urgent need to find new or improved known alternative methods for treating infections caused by MDR strains. Endolysins, despite their limitations in terms of selective activity against Gram-negative bacteria, represent an interesting and potentially effective solution, especially when combined with agents that disrupt the structure of the outer membrane of these bacteria. Structural modifications of endolysins to enhance their bactericidal potential are also worth noting. Such an alternative strategy involves fusing endolysins with components that facilitate their access to the peptidoglycan layer. For instance, the *E. coli* phage lysin Lysep3 has been fused with the endolysin-binding domain D8 from *Bacillus amyloliquefaciens*. On its own, Lysep3 cannot break through *E. coli*’s outer membrane. However, when combined with the D8 domain, it gains the ability to destroy the bacteria externally. This is because D8 weakens the outer membrane’s structure, allowing the enzyme to reach and degrade the peptidoglycan layer [74]. Other solutions of this problem are artilysin, which involves the fusion of LPS-disrupting molecules to a lysin; lysocins, which are combined endolysins and bacteriocins; innolysins, which exploit phage receptor-binding proteins (RBPs) to bind surface receptors to destabilize the outer membrane, synergy with permeabilizing chemicals; other phage enzymes, such as depolymerases, that degrade capsular polysaccharides, or even antibiotics; and finally, adding cationic compounds or antimicrobial peptides (AMPs) to the N- or C- terminus, as reviewed recently [48,75]. Importantly, the combined antimicrobial effect of phage-derived endolysin and depolymerase against biofilm-forming *Salmonella* Typhimurium was reported by Kim et al. [76]. However, studies accounting for fusions of these proteins or the combined effect of depolymerases and phage endolysins on Gram-negative bacteria are still missing.

To fully assess the potential of phage-derived enzymes in combating infections caused by antibiotic-resistant bacteria, including UPEC, it is essential to optimize their action against biofilms, conduct comprehensive safety studies, and test the efficacy of these proteins in clinical practice.

## 5. Conclusions

Interestingly, not only whole bacterial viruses, but also phage-derived proteins with lytic properties, named endolysins, have gained researchers’ interest in recent years. Endolysins seem to possess greater application potential than intact phage particles, making them very promising and effective candidates for use as alternatives to antibiotics, also in UTI treatment. The major advantages of endolysins are the following: rapid bacterial cell lysis, a low probability of developing resistance, synergistic action with other antimicrobial agents, and high efficacy in planktonic and biofilm cells. Moreover, through genetic modification of endolysins, their lytic spectrum of action may be changed. Due to the above-mentioned unique features, parental and recombinant endolysins have been applied in much research.

We observe a growing interest in both phages and their enzymes; however, the number of identified and well-characterized phage particles or lytic proteins is still too small and not sufficiently studied for them to be introduced into standard treatment. The first main problem of endolysins concerns the difficulties with their identification in phage genomes. Our analyses have shown that annotation programs can make mistakes and mark proteins as endolysins that finally turn out not to be ones. Moreover, the nomenclature of phage proteins is not unified, which introduces confusion in the marking. The second problem is a broad spectrum of endolysins, which, when applied as antimicrobial agents, could affect the patient’s microbiota and induce dysbiosis. Interesting solutions in this case can be the addition of a cell wall-binding domain to target only specific bacteria or phage RBPs. Other problems concern the difficulties with the purification of endolysins and their limited action from the outside of the cell, especially in the case of Gram-negative bacteria. The endolysin diffusion through the outer membrane of such bacteria is limited and, when administered exogenously, they are not able to reach their site of action—peptidoglycan. In general, three main approaches have recently been explored to bypass the outer membrane of Gram-negative bacteria. These include utilizing lysins with intrinsic activity stemming from a positively charged C-terminus that destabilizes the membrane, employing physical or chemical methods to disrupt membrane integrity, and applying protein engineering to obtain the fusion of endolysins to components that allow them to gain access to the peptidoglycan layer. Undoubtedly, phage endolysins still face many challenges; however, they seem to be promising and effective candidates for use against bacteria, including UPEC.

## Figures and Tables

**Table 1:** Characteristics of endolysins annotated in genomes of anti-UPEC bacteriophages.

Phage name [References]	Protein Name	Position of the Gene in Virus Genome	Gene Length (nt)	Protein Accession Number	Protein Length (aa)	Molecular Weight (kDa)	Instability Index	Isoelectric Point (pI)	Gravy	Aliphatic Index	Melting Point (Tm)	Name of the Predicted Domain *	Position of the Domain in Protein Sequence
vB_EcoM_LNA1 [27]	endolysin	77244–77708	464	self_ZKRH133	154	17.21	33.482	9.36	−0.381	81.104	47.0 °C	lyz_endolysin_autolysin (cd00737) Phage lysozyme (PF00959) Lysozyme-like (SSF53955) Endolysin (MF_04110)	7–148 13–143 1–152 1–150
590B [28]	endolysin	39903–40391	488	QVR48622.1	162	17.11	20.515	9.86	−0.125	87.531	51.0 °C	lyz_endolysin_autolysin (cd00737) Phage lysozyme (PF00959) Lysozyme-like (SSF53955)	9–146 15–138 3–149
vB_EcoS_UTEC10 [29]	endolysin	1847–2332	485	WWY66481.1	161	17.03	20.046	9.81	−0.12	88.696	50.0 °C	lyz_endolysin_autolysin (cd00737) Phage lysozyme (PF00959) Lysozyme-like (SSF53955)	9–146 15–138 3–149
vB_EcoS-phiEc3 [30]	endolysin	41533–42021	488	YP_010749568.1	162	17.05	19.526	9.81	−0.101	88.148	51.0 °C	lyz_endolysin_autolysin (cd00737) Phage lysozyme (PF00959) Lysozyme-like (SSF53955)	9–146 15–138 3–149
NTEC3 [31]	endolysin	11540–12028	488	YP_010749155.1	162	17.14	21.851	9.86	−0.137	87.531	50.0 °C	lyz_endolysin_autolysin (cd00737) Phage lysozyme (PF00959) Lysozyme-like (SSF53955)	9–146 15–138 3–149
vB_EcoS-phiEc4 [30]	endolysin	41414–41899	485	QYU43915.1	161	17.11	22.631	9.92	−0.139	88.075	50.0 °C	lyz_endolysin_autolysin (cd00737) Phage lysozyme (PF00959) Lysozyme-like (SSF53955)	9–146 15–138 3–149
VB_EcoS-Golestan [32]	endolysin	41444–41902	458	YP_009620148.1	152	16.09	17.289	9.86	−0.069	88.816	49.0 °C	lyz_endolysin_autolysin (cd00737) Phage lysozyme (PF00959) Lysozyme-like (SSF53955)	3–137 6–129 2–141
vB_EcoM_SHAK7858 [33]	endolysin	40989–41474	485	WVW77834.1	161	17.11	19.781	9.62	−0.141	84.472	50.0 °C	lyz_endolysin_autolysin (cd00737) Phage lysozyme (PF00959) Lysozyme-like (SSF53955)	8–145 14–137 4–148
vB_EcoS_ACG-M12 [34]	endolysin	41386–41871	485	YP_006987885.1	161	17.63	33.838	9.83	−0.108	99.938	49.0 °C	endolysin_R21-like (cd16900) SAR-endolysin (MF_04136) Phage lysozyme (PF00959) Lysozyme-like (SSF53955) Endolysin (MF_04110)	20–156 3–158 25–151 6–159 4–158
Killian [35]	endolysin	77692–78186	494	WEU67899.1	164	18.59	37.155	9.59	−0.37	88.049	53.0 °C	T4-like_lys (cd00735) Phage lysozyme (PF00959) Lysozyme-like (SSF53955) Endolysin (MF_04110) Endolysin (G3DSA:1.10.530.40:FF:000002)	2–160 11–141 1–161 1–159 1–164
vB_EcoM_SHAK7693 [33]	endolysin	42651–42953	302	WVW77754.1	100	10.36	21.694	10.21	0.061	96.9	49.0 °C	Phage lysozyme (PF00959) Lysozyme-like (SSF53955)	2–76 2–86
vB_EcoM_SHAK7693 [33]	endolysin	42419–42658	239	WVW77753.1	79	8.73	25.656	7.91	−0.537	70.38	49.0 °C	Phage lysozyme (PF00959) Lysozyme-like (SSF53955)	20–67 6–68
vB_EcoP-101136BS1 [36]	endolysin	19728–20186	458	QZI79903.1	152	17.06	44.325	8.83	−0.391	78.158	51.0 °C	PGRP (cd06583) N-acetylmuramoyl-L-alanine amidase-like (SSF55846) N-acetylmuramoyl-L-alanine amidase (PF01510) Endolysin (MF_04111)	14–133 11–147 11–134 2–150
vB_EcoP-101120B2 [36]	endolysin	20895–21353	458	QZI79844.1	152	17.1	44.325	8.47	−0.46	76.25	51.0 °C	PGRP (cd06583) N-acetylmuramoyl-L-alanine amidase-like (SSF55846) N-acetylmuramoyl-L-alanine amidase (PF01510) Endolysin (MF_04111)	14–133 11–147 11–134 2–150
vB_EcoP-101120B1-2 [36]	endolysin	20840–21298	458	QZI79784.1	152	17.1	44.325	8.47	−0.46	76.25	51.0 °C	PGRP (cd06583) N-acetylmuramoyl-L-alanine amidase-like (SSF55846) N-acetylmuramoyl-L-alanine amidase (PF01510) Endolysin (MF_04111)	14–133 11–147 11–134 2–150
vB_EcoP-101118UKE1 [36]	endolysin	20127–20585	458	QZI79721.1	152	17.04	44.76	7.79	−0.38	78.158	51.0 °C	PGRP (cd06583) N-acetylmuramoyl-L-alanine amidase-like (SSF55846) N-acetylmuramoyl-L-alanine amidase (PF01510) Endolysin (MF_04111)	14–133 11–147 11–134 2–150
vB_EcoP-22664UKE3-2 [36]	endolysin	21058–21516	458	QZI78611.1	152	17.12	45.137	8.47	−0.458	76.908	51.0 °C	PGRP (cd06583) N-acetylmuramoyl-L-alanine amidase-like (SSF55846) N-acetylmuramoyl-L-alanine amidase (PF01510) Endolysin (MF_04111)	14–133 11–147 11–134 2–150
vB_EcoP-22664BS1 [36]	endolysin	19873–20226	353	QZI78465.1	117	13.16	50.611	7.83	−0.531	77.436	49.0 °C	PGRP (cd06583) N-acetylmuramoyl-L-alanine amidase-like (SSF55846) N-acetylmuramoyl-L-alanine amidase (PF01510)	5–98 2–112 7–99
vB_EcoP_ACG-C91 [34]	endolysin	39340–39684	344	YP_006987811.1	114	12.75	25.157	9.51	−0.333	72.632	52.0 °C	Peptidase M15 (PF08291) Hedgehog/DD-peptidase (SSF55166)	4–106 5–107
vB_EcoM_SHAK7163 [33]	endolysin	39749–40093	344	WVW77668.1	114	12.59	29.711	9.32	−0.348	71.754	50.0 °C	Peptidase M15 (PF08291) Hedgehog/DD-peptidase (SSF55166)	5–106 5–107
vB-EcoP-KMB14 [37]	endolysin	38777–39121	344	WPK27911.1	114	12.65	40.639	9.35	−0.392	67.544	49.0 °C	Peptidase M15 (PF08291) Hedgehog/DD-peptidase (SSF55166)	4–106 5–108
vB_EcoM_SHAK7704 [33]	endolysin	3327–3671	344	self_RMKP5	114	12.65	25.787	9.32	−0.401	70.088	51.0 °C	Peptidase M15 (PF08291) Hedgehog/DD-peptidase (SSF55166)	5–106 5–107
MLP1 [38]	endolysin	32790–33185	395	YP_010844467.1	131	14.96	47.821	8.79	−0.303	84.122	57.0 °C	L-Ala-D-Glu_peptidase_like (cd14845) Hedgehog/DD-peptidase (SSF55166) D-alanyl-D-alanine carboxypeptidase (PF13539)	9–130 4–129 58–110
vB-EcoM-KMB43 [37]	endolysin	55240–55629	389	WQN06429.1	129	14.72	12.666	9.3	−0.412	79.922	53.0 °C	L-Ala-D-Glu_peptidase_like (cd14845) Hedgehog/DD-peptidase (SSF55166) D-alanyl-D-alanine carboxypeptidase (PF13539)	11–120 2–120 55–120
vB_EcoP_EG1 [39]	internal virion protein with endolysin domain	30249–34223	3974	YP_009795842.1	1324	145.33	36.856	6.58	−0.397	78.52	41.0 °C	Transglycosylase SLT domain (PF01464) Peptidoglycan transglycosylase gp16. (MF_04121) Lysozyme-like (SSF53955) LT-like (cd00254)	15–127 2–1314 5–146 28–144
vB_EcoM_LNA4 [27]	endolysin	21617–22171	554	self_PFXJ59	184	20.08	23.118	9.3	−0.335	88.098	53.0 °C	Lysozyme-like (SSF53955) Chitinase class I (PF00182)	3–182 41–146
vB_EcoM_LNA4 [27]	endolysin	117557–118129	572	self_PFXJ204	190	21.65	30.802	9.67	−0.374	81.105	41.0 °C	Cell Wall Hydrolase (PF07486)	70–181
Killian [35]	endolysin	54687–56504	1817	WEU67851.1	605	67.92	35.65	6.61	−0.302	82.033	44.0 °C	anaerobic ribonucleoside-triphosphate reductase (TIGR02487) RNR_III (cd01675) PFL-like glycyl radical enzymes (SSF51998) Anaerobic ribonucleoside-triphosphate reductase (PF13597)	21–604 21–584 26–585 21–604
vB_Ec_ZCEC14 [40]	endolysin	13508–14425	917	UVD33329.1	305	35.56	28.885	8.61	−0.448	85.967	42.0 °C	T4 RNase H, C terminal (PF09293) PIN_53EXO-like (cd00008) PIN domain-like (SSF88723) H3TH_T4-like (cd09899) 5′-3′ exonuclease, N-terminal resolvase-like domain (PF02739) 5′ to 3′ exonuclease, C-terminal subdomain (SSF47807)	183–305 17–180 13–179 184–259 51–174 183–305
Killian [35]	endolysin	163120–164037	917	WEU68017.1	305	35.56	28.885	8.61	−0.448	85.967	42.0 °C	T4 RNase H, C terminal (PF09293) PIN_53EXO-like (cd00008) PIN domain-like (SSF88723) H3TH_T4-like (cd09899) 5′-3′ exonuclease, N-terminal resolvase-like domain (PF02739) 5′ to 3′ exonuclease, C-terminal subdomain (SSF47807)	183–305 17–180 13–179 184–259 51–174 183–305
vB_Ec_ZCEC14 [40]	endolysin	51539–52219	680	UVD33388.1	226	25.57	45.489	4.43	−0.378	91.947	48.0 °C	Bacteriophage T4, inh (IPR016594)	1–226
Killian [35]	endolysin	46919–47182	263	WEU67833.1	87	10.24	48.485	4.21	−0.197	74.943	40.0 °C	-	-
vB_EcoM_SHAK7163 [33]	internal virion protein with endolysin domain	31648–34968	3320	WVW77660.1	1106	121.66	35.115	5.61	−0.423	79.322	42.0 °C	-	-
vB_EcoM_SHAK7704 [33]	internal virion protein with endolysin domain	39690–42998	3308	self_RMKP62	1102	121.26	35.421	5.69	−0.429	78.63	42.0 °C	-	-
vB_EcoM_SHAK7854 [33]	internal virion protein with endolysin domain	32030–35917	3887	self_DNYN53	1295	140.9	37.494	5.67	−0.396	79.012	41.0 °C	-	-
vB_EcoM_SHAK9454 [33]	internal virion protein with endolysin domain	32160–36047	3887	self_ZENB53	1295	140.83	37.467	5.87	−0.39	78.942	41.0 °C	-	-
vB_EcoP_LNA3 [27]	internal virion protein with endolysin domain	5943–9830	3887	self_GXIJ6	1295	140.96	36.837	5.68	−0.399	79.459	41.0 °C	-	-
vB_EcoP_LNA5 [27]	internal virion protein with endolysin domain	23530–27417	3887	self_TCEH45	1295	140.81	37.737	5.93	−0.398	77.892	41.0 °C	-	-
vB_Ec_ZCEC14 [40]	endolysin	127670–127933	263	UVD33512.1	87	10.29	48.816	4.3	−0.277	74.943	40.0 °C	-	-

* Endolysins are arranged based on the type of domain they possess.

## Supplementary Materials

The following supporting information can be downloaded at https://www.mdpi.com/article/10.3390/v17040560/s1: Supplementary Table S1. Data obtained by InterProScan version 5.72-103 software, giving an overview of the domains identified within the annotated endolysins. Supplementary Table S2. The list of AreMP peptides identified within sequences of putative endolysins. Supplementary Table S3. Characteristics of the proteins identified by phylogenetic analysis as closely related to the identified endolysins. Supplementary Table S4. Data obtained by InterProScan version 5.72-103 software, giving an overview of the domains identified within the proteins found by phylogenetic analysis as closely related to the tested endolysins. Supplementary Table S5. The list of AMP peptides found within sequences of the proteins identified by phylogenetic analysis as closely related to the tested endolysins. Supplementary Table S6. Data providing an overview of the size, genome position, stability, and domains identified within the endolysins derived from model phages λ, T4, T5, T7, and SP6. Supplementary Table S7. Results of the comparative analysis of all endolysin sequences tested in this work and those derived from model phages λ, T4, T5, T7, and SP6.

## References

[1] Gondil V.S., Chhibber S. (2021). Bacteriophage and Endolysin Encapsulation Systems: A Promising Strategy to Improve Therapeutic Outcomes. Front. Pharmacol..

[2] Abdelrahman F., Easwaran M., Daramola O.I., Ragab S., Lynch S., Oduselu T.J., Khan F.M., Ayobami A., Adnan F., Torrents E. (2021). Phage-Encoded Endolysins. Antibiotics.

[3] Principi N., Silvestri E., Esposito S. (2019). Advantages and Limitations of Bacteriophages for the Treatment of Bacterial Infections. Front. Pharmacol..

[4] Fischetti V.A. (2018). Development of Phage Lysins as Novel Therapeutics: A Historical Perspective. Viruses.

[5] Theuretzbacher U., Piddock L.J.V. (2019). Non-Traditional Antibacterial Therapeutic Options and Challenges. Cell Host Microbe.

[6] Love M.J., Abeysekera G.S., Muscroft-Taylor A.C., Billington C., Dobson R.C.J. (2020). On the Catalytic Mechanism of Bacteriophage Endolysins: Opportunities for Engineering. Biochim. Biophys. Acta Proteins Proteom..

[7] Li X., Zhang C., Wei F., Yu F., Zhao Z. (2021). Bactericidal Activity of a Holin-Endolysin System Derived from Vibrio Alginolyticus Phage HH109. Microb. Pathog..

[8] Nazir A., Xu X., Liu Y., Chen Y. (2023). Phage Endolysins: Advances in the World of Food Safety. Cells.

[9] Khan F.M., Chen J.-H., Zhang R., Liu B. (2023). A Comprehensive Review of the Applications of Bacteriophage-Derived Endolysins for Foodborne Bacterial Pathogens and Food Safety: Recent Advances, Challenges, and Future Perspective. Front. Microbiol..

[10] Oliveira H., São-José C., Azeredo J. (2018). Phage-Derived Peptidoglycan Degrading Enzymes: Challenges and Future Prospects for In Vivo Therapy. Viruses.

[11] Schmelcher M., Donovan D.M., Loessner M.J. (2012). Bacteriophage Endolysins as Novel Antimicrobials. Future Microbiol..

[12] Microbial Products for Health, Environment and Agriculture. https://www.researchgate.net/publication/354735414_Microbial_Products_for_Health_Environment_and_Agriculture.

[13] Liu H., Hu Z., Li M., Yang Y., Lu S., Rao X. (2023). Therapeutic Potential of Bacteriophage Endolysins for Infections Caused by Gram-Positive Bacteria. J. Biomed. Sci..

[14] Lai W.C.B., Chen X., Ho M.K.Y., Xia J., Leung S.S.Y. (2020). Bacteriophage-Derived Endolysins to Target Gram-Negative Bacteria. Int. J. Pharm..

[15] Oechslin F., Zhu X., Dion M.B., Shi R., Moineau S. (2022). Phage Endolysins Are Adapted to Specific Hosts and Are Evolutionarily Dynamic. PLoS Biol..

[16] Haddad Kashani H., Schmelcher M., Sabzalipoor H., Seyed Hosseini E., Moniri R. (2018). Recombinant Endolysins as Potential Therapeutics against Antibiotic-Resistant Staphylococcus Aureus: Current Status of Research and Novel Delivery Strategies. Clin. Microbiol. Rev..

[17] Rahman M.U., Wang W., Sun Q., Shah J.A., Li C., Sun Y., Li Y., Zhang B., Chen W., Wang S. (2021). Endolysin, a Promising Solution against Antimicrobial Resistance. Antibiotics.

[18] São-José C. (2018). Engineering of Phage-Derived Lytic Enzymes: Improving Their Potential as Antimicrobials. Antibiotics.

[19] Ghose C., Euler C.W. (2020). Gram-Negative Bacterial Lysins. Antibiotics.

[20] Gontijo M.T.P., Jorge G.P., Brocchi M. (2021). Current Status of Endolysin-Based Treatments against Gram-Negative Bacteria. Antibiotics.

[21] Gontijo M.T.P., Vidigal P.M.P., Lopez M.E.S., Brocchi M. (2021). Bacteriophages That Infect Gram-Negative Bacteria as Source of Signal-Arrest-Release Motif Lysins. Res. Microbiol..

[22] Zalewska-Piątek B., Piątek R. (2020). Phage Therapy as a Novel Strategy in the Treatment of Urinary Tract Infections Caused by *E. coli*. Antibiotics.

[23] Melican K., Sandoval R.M., Kader A., Josefsson L., Tanner G.A., Molitoris B.A., Richter-Dahlfors A. (2011). Uropathogenic Escherichia Coli P and Type 1 Fimbriae Act in Synergy in a Living Host to Facilitate Renal Colonization Leading to Nephron Obstruction. PLoS Pathog..

[24] Chen L., Wen Y. (2011). The Role of Bacterial Biofilm in Persistent Infections and Control Strategies. Int. J. Oral Sci..

[25] Lila A.S.A., Rajab A.A.H., Abdallah M.H., Rizvi S.M.D., Moin A., Khafagy E.-S., Tabrez S., Hegazy W.A.H. (2023). Biofilm Lifestyle in Recurrent Urinary Tract Infections. Life.

[26] Necel A., Bloch S., Topka-Bielecka G., Janiszewska A., Łukasiak A., Nejman-Faleńczyk B., Węgrzyn G. (2022). Synergistic Effects of Bacteriophage vB_Eco4-M7 and Selected Antibiotics on the Biofilm Formed by Shiga Toxin-Producing *Escherichia coli*. Antibiotics.

[27] Ngiam L., Schembri M.A., Weynberg K., Guo J. (2021). Bacteriophage Isolated from Non-Target Bacteria Demonstrates Broad Host Range Infectivity against Multidrug-Resistant Bacteria. Environ. Microbiol..

[28] Chaudhary N., Maurya R.K., Singh D., Mohan B., Taneja N. (2022). Genome Analysis and Antibiofilm Activity of Phage 590B against Multidrug-Resistant and Extensively Drug-Resistant Uropathogenic *Escherichia coli* Isolates, India. Pathogens.

[29] Rajab A.A.H., Fahmy E.K., Esmaeel S.E., Yousef N., Askoura M. (2024). In Vitro and in Vivo Assessment of the Competence of a Novel Lytic Phage vB_EcoS_UTEC10 Targeting Multidrug Resistant *Escherichia coli* with a Robust Biofilm Eradication Activity. Microb. Pathog..

[30] González-Villalobos E., Ribas-Aparicio R.M., Montealegre G.E.R., Belmont-Monroy L., Ortega-García Y., Aparicio-Ozores G., Balcázar J.L., Eslava-Campos C.A., Hernández-Chiñas U., Molina-López J. (2021). Isolation and Characterization of Novel Bacteriophages as a Potential Therapeutic Option for *Escherichia coli* Urinary Tract Infections. Appl. Microbiol. Biotechnol..

[31] Chaudhary N., Singh D., Maurya R.K., Mohan B., Mavuduru R.S., Taneja N. (2022). Whole Genome Sequencing and in Vitro Activity Data of *Escherichia* Phage NTEC3 against Multidrug-Resistant Uropathogenic and Extensively Drug-Resistant Uropathogenic *E. coli* Isolates. Data Brief.

[32] Yazdi M., Bouzari M., Ghaemi E.A., Shahin K. (2020). Isolation, Characterization and Genomic Analysis of a Novel Bacteriophage VB_EcoS-Golestan Infecting Multidrug-Resistant *Escherichia coli* Isolated from Urinary Tract Infection. Sci. Rep..

[33] Asgharzadeh Kangachar S., Logel D.Y., Trofimova E., Zhu H.X., Zaugg J., Schembri M.A., Weynberg K.D., Jaschke P.R. (2024). Discovery and Characterisation of New Phage Targeting Uropathogenic *Escherichia coli*. Virology.

[34] Chibeu A., Lingohr E.J., Masson L., Manges A., Harel J., Ackermann H.-W., Kropinski A.M., Boerlin P. (2012). Bacteriophages with the Ability to Degrade Uropathogenic *Escherichia coli* Biofilms. Viruses.

[35] Khunti P., Chantakorn K., Tantibhadrasapa A., Htoo H.H., Thiennimitr P., Nonejuie P., Chaikeeratisak V.A. (2023). Novel Coli Myophage and Antibiotics Synergistically Inhibit the Growth of the Uropathogenic *E. coli* Strain CFT073 in Stoichiometric Niches. Microbiol. Spectr..

[36] Loose M., Sáez Moreno D., Mutti M., Hitzenhammer E., Visram Z., Dippel D., Schertler S., Tišáková L.P., Wittmann J., Corsini L. (2021). Natural Bred Ε2-Phages Have an Improved Host Range and Virulence against Uropathogenic *Escherichia coli* over Their Ancestor Phages. Antibiotics.

[37] Markusková B., Elnwrani S., Andrezál M., Sedláčková T., Szemes T., Slobodníková L., Kajsik M., Drahovská H. (2024). Characterization of Bacteriophages Infecting Multidrug-Resistant Uropathogenic *Escherichia coli* Strains. Arch. Virol..

[38] Vera-Mansilla J., Sánchez P., Silva-Valenzuela C.A., Molina-Quiroz R.C. (2022). Isolation and Characterization of Novel Lytic Phages Infecting Multidrug-Resistant *Escherichia coli*. Microbiol. Spectr..

[39] Gu Y., Xu Y., Xu J., Yu X., Huang X., Liu G., Liu X. (2019). Identification of Novel Bacteriophage vB_EcoP-EG1 with Lytic Activity against Planktonic and Biofilm Forms of Uropathogenic *Escherichia coli*. Appl. Microbiol. Biotechnol..

[40] Ismael N.M., Azzam M., Abdelmoteleb M., El-Shibiny A. (2024). Phage vB_Ec_ZCEC14 to Treat Antibiotic-Resistant Escherichia Coli Isolated from Urinary Tract Infections. Virol. J..

[41] Slobodníková L., Markusková B., Kajsík M., Andrezál M., Straka M., Liptáková A., Drahovská H. (2021). Characterization of Anti-Bacterial Effect of the Two New Phages against Uropathogenic *Escherichia coli*. Viruses.

[42] Bouras G., Nepal R., Houtak G., Psaltis A.J., Wormald P.-J., Vreugde S. (2023). Pharokka: A Fast Scalable Bacteriophage Annotation Tool. Bioinformatics.

[43] Cock P.J.A., Antao T., Chang J.T., Chapman B.A., Cox C.J., Dalke A., Friedberg I., Hamelryck T., Kauff F., Wilczynski B. (2009). Biopython: Freely Available Python Tools for Computational Molecular Biology and Bioinformatics. Bioinformatics.

[44] Jung F., Frey K., Zimmer D., Mühlhaus T. (2023). DeepSTABp: A Deep Learning Approach for the Prediction of Thermal Protein Stability. Int. J. Mol. Sci..

[45] Ikai A. (1980). Thermostability and Aliphatic Index of Globular Proteins. J. Biochem..

[46] Jones P., Binns D., Chang H.-Y., Fraser M., Li W., McAnulla C., McWilliam H., Maslen J., Mitchell A., Nuka G. (2014). InterProScan 5: Genome-Scale Protein Function Classification. Bioinformatics.

[47] Blum M., Chang H.-Y., Chuguransky S., Grego T., Kandasaamy S., Mitchell A., Nuka G., Paysan-Lafosse T., Qureshi M., Raj S. (2021). The InterPro Protein Families and Domains Database: 20 Years on. Nucleic Acids Res..

[48] Murray E., Draper L.A., Ross R.P., Hill C. (2021). The Advantages and Challenges of Using Endolysins in a Clinical Setting. Viruses.

[49] Fenton M., Ross P., McAuliffe O., O’Mahony J., Coffey A. (2010). Recombinant Bacteriophage Lysins as Antibacterials. Bioeng. Bugs.

[50] Marchler-Bauer A., Bo Y., Han L., He J., Lanczycki C.J., Lu S., Chitsaz F., Derbyshire M.K., Geer R.C., Gonzales N.R. (2017). CDD/SPARCLE: Functional Classification of Proteins via Subfamily Domain Architectures. Nucleic Acids Res..

[51] Lu S., Wang J., Chitsaz F., Derbyshire M.K., Geer R.C., Gonzales N.R., Gwadz M., Hurwitz D.I., Marchler G.H., Song J.S. (2020). CDD/SPARCLE: The Conserved Domain Database in 2020. Nucleic Acids Res..

[52] Wang J., Chitsaz F., Derbyshire M.K., Gonzales N.R., Gwadz M., Lu S., Marchler G.H., Song J.S., Thanki N., Yamashita R.A. (2023). The Conserved Domain Database in 2023. Nucleic Acids Res..

[53] Turnau K., Fiałkowska E., Ważny R., Rozpądek P., Tylko G., Bloch S., Nejman-Faleńczyk B., Grabski M., Węgrzyn A., Węgrzyn G. (2021). Extraordinary Multi-Organismal Interactions Involving Bacteriophages, Bacteria, Fungi, and Rotifers: Quadruple Microbial Trophic Network in Water Droplets. Int. J. Mol. Sci..

[54] Altenhoff A.M., Levy J., Zarowiecki M., Tomiczek B., Warwick Vesztrocy A., Dalquen D.A., Müller S., Telford M.J., Glover N.M., Dylus D. (2019). OMA Standalone: Orthology Inference among Public and Custom Genomes and Transcriptomes. Genome Res..

[55] Minh B.Q., Schmidt H.A., Chernomor O., Schrempf D., Woodhams M.D., von Haeseler A., Lanfear R. (2020). IQ-TREE 2: New Models and Efficient Methods for Phylogenetic Inference in the Genomic Era. Mol. Biol. Evol..

[56] Sievers F., Wilm A., Dineen D., Gibson T.J., Karplus K., Li W., Lopez R., McWilliam H., Remmert M., Söding J. (2011). Fast, Scalable Generation of High-Quality Protein Multiple Sequence Alignments Using Clustal Omega. Mol. Syst. Biol..

[57] Tamura K., Stecher G., Kumar S. (2021). MEGA11: Molecular Evolutionary Genetics Analysis Version 11. Mol. Biol. Evol..

[58] Rodríguez-Rubio L., Martínez B., Donovan D.M., Rodríguez A., García P. (2013). Bacteriophage virion-associated peptidoglycan hydrolases: Potential new enzybiotics. Crit. Rev. Microbiol..

[59] Zhao A., Sun J., Liu Y. (2023). Understanding Bacterial Biofilms: From Definition to Treatment Strategies. Front. Cell. Infect. Microbiol..

[60] Gondil V.S., Harjai K., Chhibber S. (2020). Endolysins as Emerging Alternative Therapeutic Agents to Counter Drug-Resistant Infections. Int. J. Antimicrob. Agents.

[61] Soontarach R., Srimanote P., Voravuthikunchai S.P., Chusri S. (2024). Antibacterial and Anti-Biofilm Efficacy of Endolysin LysAB1245 against a Panel of Important Pathogens. Pharmaceuticals.

[62] Carratalá J.V., Ferrer-Miralles N., Garcia-Fruitós E., Arís A. (2024). LysJEP8: A Promising Novel Endolysin for Combating Multidrug-Resistant Gram-Negative Bacteria. Microb. Biotechnol..

[63] Guo M., Feng C., Ren J., Zhuang X., Zhang Y., Zhu Y., Dong K., He P., Guo X., Qin J. (2017). A Novel Antimicrobial Endolysin, LysPA26, against Pseudomonas Aeruginosa. Front. Microbiol..

[64] Vasina D.V., Antonova N.P., Grigoriev I.V., Yakimakha V.S., Lendel A.M., Nikiforova M.A., Pochtovyi A.A., Remizov T.A., Usachev E.V., Shevlyagina N.V. (2021). Discovering the Potentials of Four Phage Endolysins to Combat Gram-Negative Infections. Front. Microbiol..

[65] Kajsikova M., Kajsik M., Bocanova L., Papayova K., Drahovska H., Bukovska G. (2024). Endolysin EN572-5 as an Alternative to Treat Urinary Tract Infection Caused by Streptococcus Agalactiae. Appl. Microbiol. Biotechnol..

[66] Fursov M.V., Abdrakhmanova R.O., Antonova N.P., Vasina D.V., Kolchanova A.D., Bashkina O.A., Rubalsky O.V., Samotrueva M.A., Potapov V.D., Makarov V.V. (2020). Antibiofilm Activity of a Broad-Range Recombinant Endolysin LysECD7: In Vitro and In Vivo Study. Viruses.

[67] Euler C.W., Raz A., Hernandez A., Serrano A., Xu S., Andersson M., Zou G., Zhang Y., Fischetti V.A., Li J. (2023). PlyKp104, a Novel Phage Lysin for the Treatment of Klebsiella Pneumoniae, Pseudomonas Aeruginosa, and Other Gram-Negative ESKAPE Pathogens. Antimicrob. Agents Chemother..

[68] Yan G., Yang R., Fan K., Dong H., Gao C., Wang S., Yu L., Cheng Z., Lei L. (2019). External Lysis of *Escherichia coli* by a Bacteriophage Endolysin Modified with Hydrophobic Amino Acids. AMB Express.

[69] Hasan M., Kim J., Liao X., Ding T., Ahn J. (2024). Antibacterial Activity of Bacteriophage-Encoded Endolysins against Planktonic and Biofilm Cells of Pathogenic *Escherichia coli*. Microb. Pathog..

[70] Park D.-W., Park J.-H. (2020). Characterization of Endolysin LysECP26 Derived from rV5-like Phage vB_EcoM-ECP26 for Inactivation of *Escherichia coli* O157:H7. J. Microbiol. Biotechnol..

[71] Oh M., Cevallos-Urena A., Kim B.S. (2024). Bacteriophages PECP14, PECP20, and Their Endolysins as Effective Biocontrol Agents for *Escherichia coli* O157:H7 and Other Foodborne Pathogens. Int. J. Food Microbiol..

[72] Chu D., Lan J., Liang L., Xia K., Li L., Yang L., Liu H., Zhang T. (2024). The Antibacterial Activity of a Novel Highly Thermostable Endolysin, LysKP213, against Gram-Negative Pathogens Is Enhanced When Combined with Outer Membrane Permeabilizing Agents. Front. Microbiol..

[73] Hwang Y.J., Jo J., Kim E., Yoon H., Hong H., Kim M.S., Myung H. (2022). Motility Increase of Adherent Invasive *Escherichia coli* (AIEC) Induced by a Sub-Inhibitory Concentration of Recombinant Endolysin LysPA90. Front. Microbiol..

[74] Wang S., Gu J., Lv M., Guo Z., Yan G., Yu L., Du C., Feng X., Han W., Sun C. (2017). The antibacterial activity of *E. coli* bacteriophage lysin lysep3 is enhanced by fusing the *Bacillus amyloliquefaciens* bacteriophage endolysin binding domain D8 to the C-terminal region. J. Microbiol..

[75] Sisson H.M., Jackson S.A., Fagerlund R.D., Warring S.L., Fineran P.C. (2024). Gram-negative endolysins: Overcoming the outer membrane obstacle. Curr. Opin. Microbiol..

[76] Kim J., Wang J., Ahn J. (2023). Combined antimicrobial effect of phage-derived endolysin and depolymerase against biofilm-forming *Salmonella* Typhimurium. Biofouling.

